# Inefficient Metabolism of the Human Milk Oligosaccharides Lacto-*N*-tetraose and Lacto-*N*-neotetraose Shifts *Bifidobacterium longum* subsp. *infantis* Physiology

**DOI:** 10.3389/fnut.2018.00046

**Published:** 2018-05-30

**Authors:** Ezgi Özcan, David A. Sela

**Affiliations:** ^1^Department of Food Science, University of Massachusetts, Amherst, MA, United States; ^2^Department of Microbiology, University of Massachusetts, Amherst, MA, United States; ^3^Department of Microbiology and Physiological Systems and Center for Microbiome Research, University of Massachusetts Medical School, Worcester, MA, United States

**Keywords:** bifidobacteria, human milk oligosaccharides, lacto-*N*-tetraose, lacto-*N*-neotetraose, host-microbial interactions, microbiota

## Abstract

Human milk contains a high concentration of indigestible oligosaccharides, which likely mediated the coevolution of the nursing infant with its gut microbiome. Specifically, *Bifidobacterium longum* subsp. *infantis* (*B. infantis*) often colonizes the infant gut and utilizes these human milk oligosaccharides (HMOs) to enrich their abundance. In this study, the physiology and mechanisms underlying *B. infantis* utilization of two HMO isomers lacto-*N*-tetraose (LNT) and lacto-*N*-neotetraose (LNnT) was investigated in addition to their carbohydrate constituents. Both LNT and LNnT utilization induced a significant shift in the ratio of secreted acetate to lactate (1.7–2.0) in contrast to the catabolism of their component carbohydrates (~1.5). Inefficient metabolism of LNnT prompts *B. infantis* to shunt carbon toward formic acid and ethanol secretion. The global transcriptome presents genomic features differentially expressed to catabolize these two HMO species that vary by a single glycosidic linkage. Furthermore, a measure of strain-level variation exists between *B. infantis* isolates. Regardless of strain, inefficient HMO metabolism induces the metabolic shift toward formic acid and ethanol production. Furthermore, bifidobacterial metabolites reduced LPS-induced inflammation in a cell culture model. Thus, differential metabolism of milk glycans potentially drives the emergent physiology of host-microbial interactions to impact infant health.

## Introduction

Breastfeeding is critical for infant development and health in the absence of formula milk substitutes. Human milk contains a high concentration of indigestible oligosaccharides, as well as other nutritive molecules that promote growth experienced early in life (1–5). Human milk oligosaccharides (HMOs), are indigestible carbohydrates soluble in human milk, and are composed of five monosaccharides: D-glucose (Glc), D-galactose (Gal), *N*-acetylglucosamine (GlcNAc), L-fucose (Fuc), and *N*-acetylneuraminic acid (Neu5Ac or sialic acid) with a varying degree of polymerization and branching (6–9). These oligosaccharides are not directly metabolized by the infant; however, commensal bifidobacteria have coevolved within the nursing infant gut to utilize HMO (10, 11).

*Bifidobacterium longum* subsp. *infantis* (*B. infantis*) colonizes the nursing infant and is typically overrepresented within the infant gut microbiome (12–15). The *B*. *infantis* genome encodes an array of glycosyl hydrolases, oligosaccharide transporters, and catabolic enzymes that enable HMO utilization (10, 16–19). Both glycosyl hydrolases and membrane-spanning transporters feed milk oligosaccharides and their derivatives into the bifidobacterial fructose-6-phosphate phosphoketolase (F6PPK) central fermentative pathway. The F6PPK is believed to be unique to the genus *Bifidobacterium*, which generates ATP from hexoses via substrate-level phosphorylation resulting in the secretion of endproducts to recycle co-factors (20–23). Bifidobacteria initially converts one mole of fructose-6-phosphate to one mole of erythrose 4-phosphate and one mole of acetyl-phosphate via F6PPK (EC 4.1.2.22). A transaldolase (EC 2.2.1.2) and transketolase (EC 2.2.1.1) converts erythrose 4-phosphate and one mole of fructose-6-phosphate to two moles of xylulose-5-phosphate, which are converted into two moles of acetyl-phosphate and glyceraldehyde 3-phosphate via xylulose-5-phosphate phosphoketolase activity (EC 4.1.2.9). Acetyl-phosphate is dephosphorylated into acetate by an acetate kinase (EC 2.7.2.1), accompanied by a single ATP per acetyl-phosphate (24). In addition, glyceraldehyde 3-phosphate is oxidized to pyruvate accompanied by the production of a single ATP. Pyruvate is converted to lactate by lactate dehydrogenase (EC 1.1.1.27) along with recycling NAD^+^ from NADH (25). For every 2 moles of hexose entering the F6PPK pathway, 3 moles of acetate and 2 moles of lactate are produced (i.e., ratio of 1.5). Based on transcriptomic evidence, it is likely that *B. infantis* catabolizes HMO-derived monosaccharides through the F6PPK pathway to generate ATP (26). This potentially links bifidobacterial physiology (i.e., flux through the F6PPK pathway) with infant nutritional and health outcomes as *B. infantis* benefits their developing host (27, 28).

In general, all *B. infantis* strains examined to date efficiently utilizes HMOs pooled from several donor mothers with the exception of one (29–33, 34). The tetrasaccharides lacto-*N*-tetraose (LNT) and lacto-*N*-neotetraose (LNnT) are highly abundant oligosaccharides secreted in human milk (2, 6). LNT (Galβ1-3GlcNAcβ1-3Galβ1-4Glc) is classified as a type I HMO, which incorporates lactosyl coupled to a lacto-*N*-biose (LNB) (Galβ1-3GlcNAc) residue. In contrast, LNnT (Galβ1-4GlcNAcβ1-3Galβ1-4Glc) is an isomer of LNT and classified as a type II oligosaccharide, linking the terminal lactosyl with *N*-acetyllactosamine (LacNAc) (Galβ1-4GlcNAc). These isomers are identical aside from a sole glycosidic linkage (i.e., β1-3 vs. β1-4) thus leading to the hypothesis that this structural variation is responsible for differential phenotypes in bifidobacterial utilization of these major HMOs.

## Materials and methods

### Bacterial strains and propagation

Bacterial strains used in this study are summarized in Table 1. Bifidobacterial strains were propagated in De Man Rogosa Sharp (MRS, Oxoid, Hampshire, England) medium supplemented with 0.05% (wt/v) L-cysteine hydrochloride (Sigma-Aldrich, St. Louis, MO) (35) at 37°C under anaerobic conditions (Coy Laboratory Products, Grass Lake, MI). Bacterial strains were routinely verified using the bifidobacterial-specific phosphoketolase assay (36) and bifidobacterial-specific PCR targeting the 16S rRNA gene sequence using the previously developed Bif164-F (5′-GGGTGGTAATGCCGGATG-3′) and Bif662-R 5′-CCACCGTTACACCGGGAA-3′) (37). In addition, the PCR-based *Bifidobacterium longum*/*infantis* ratio analysis (BLIR) was performed to verify subspecies as previously described (38).

**Table 1:** List of strains used in this study^a^.

**Strain**	**Species**	**Origin**
ATCC 15697	*B. longum* subsp. *infantis*	Human infant feces
UMA299	*B. longum* subsp. *infantis*	Human infant feces
UMA300	*B. longum* subsp. *infantis*	Human infant feces
UMA301	*B. longum* subsp. *infantis*	Human infant feces

a *UMA, University of Massachusetts Amherst Culture Collection; ATCC, American Type Culture Collection*.

### Microplate growth assay

In order to evaluate growth phenotypes in a 96-well format, overnight cultures were inoculated 1% (v/v) to modified MRS media (mMRS; a defined carbohydrate substrate and no acetate). Carbohydrate substrates used in this study include glucose (Sigma-Aldrich Co. St. Louis, MO), galactose (Sigma-Aldrich Co. St. Louis, MO), lactose (Sigma-Aldrich Co. St. Louis, MO), L-fucose (Sigma-Aldrich Co. St. Louis, MO), *N*-acetylglucosamine (GlcNAc) (Tokyo Chemical Industry Co, Tokyo Japan), lacto-*N*-tetraose (LNT) (Elicityl-oligotech, Crolles, France), and lacto-*N*-neotetraose (LNnT) (Elicityl-oligotech, Crolles, France) at a final concentration of 2% (wt/v) as the sole carbon source. Carbohydrate sources were incorporated into culture media in non-limiting concentrations. The growth assay was conducted anaerobically at 37°C for 48 h by assessing optical density at 600 nm (OD_600 nm_) in an automated PowerWave HT microplate spectrophotometer (BioTek Instruments, Inc. Winooski, VT) placed within the anaerobic chamber. Each strain was evaluated in biological triplicates with three technical replicates. Inoculated mMRS media in the absence of carbohydrate substrates served as the negative control. Bacterial growth kinetics were calculated using Wolfram Mathematica 10.3 Student Edition with the equation below as described in Dai et al. (39).

$$\Delta OD\left(t\right)=\Delta ODasym\{\frac{1}{1+\exp\left[ktc-t\right]}-\frac{1}{1+\exp\left[ktc\right]}\}$$

ΔOD_asym_ is the growth level at stationary phase with k representing the growth rate and tc is the inflection point indicating the time to reach the highest growth rate.

### Characterization of microbial metabolic endproducts

Endproducts from bacterial fermentation were quantitated by HPLC. Bacterial strains were initially propagated as described above. Cell-free supernatants from microplate growths were obtained at early stationary phase and filtered through a 0.22 μm filter (Sartorius Corp, Bohemia, NY) following centrifugation and stored at −20°C until further analysis. Organic acids were quantified using a Schimadzu HPLC system equipped with a Refractive Index Detector 20A, (Schimadzu Corp., Kyoto, Japan). Separation was carried out using an Aminex HPX-87H column (7.8 mm ID × 300 mm, Bio Rad Laboratories, Hercules, CA) at 30°C in a mobile phase of 5 mM H_2_SO_4_ at flow rate of 0.6 ml/min with 20 μl of injection volume. Standards including organic acids (i.e., acetic acid, lactic acid, formic acid), ethanol, and carbohydrates (i.e., glucose, galactose, lactose, and GlcNAc) were acquired from Sigma-Aldrich Co. (St. Louis, MO). Metabolite concentrations were calculated from standard curves derived from external standards for six concentrations (0.5, 1, 5, 10, 20, and 50 mM). Metabolite profiling was carried out in triplicate and each measurement was performed in duplicate. The metabolite profiling for each strain subsisting on the panel of carbohydrates were analyzed using MetaboAnalyst 3.0 (http://www.metaboanalyst.ca) (40). The sugar consumption in percentages was calculated by dividing the amount of mono- and di-saccharide consumed after fermentation by the concentration of carbohydrate source prior to fermentation. The carbon recovery in percentages was calculated by dividing the total amount of carbon recovered in the metabolites by the total amount of carbon present in pre-fermentation minus total carbon of carbohydrate source after fermentation.

### Quantitative real-time PCR analysis

*B. infantis* gene expression was performed by quantitative real-time PCR (qRT-PCR) on a relative basis. One ml samples were harvested at mid-exponential phase (OD_600 nm_ ~ 0.4–0.6 varied depending on carbohydrate source), pelleted at 12,000 × g for 2 min, and stored in 1 ml Ambion RNAlater (Life Technologies, Carlsbad, CA). RNA extraction and cDNA conversion was performed as previously described (26). Briefly, samples were centrifuged at 12,000 × g for 2 min to collect the cell pellet. The pellet was washed twice with PBS buffer to remove residual RNAlater and centrifuged at 12,000 × g for 2 min. Total RNA was extracted using Ambion RNAqeous-Mini kit (LifeTechnologies, Carlsbad, CA) according to the manufacturer's instructions. Cells suspended in lysis buffer were transferred to the Lysing Matrix E tubes (MP Biomedicals LLC, Solon, Ohio) to disrupt cell walls through beadbeating at 5.5 m/s for 30 s twice using FastPrep 24 bead beader (MP Biomedicals, Santa Ana, CA). Total RNA was eluted in 50 μl of EB solution and immediately subjected to DNase treatment with the Ambion Turbo DNA-free (Invitrogen, Vilnius, Lithuania) using 1 μl of DNase I for 30 min. Subsequently, total RNA was converted to cDNA using the High Capacity cDNA Reverse Transcription Kit (Applied Biosystems, Carlsbad, CA) according to the manufacturer's instructions. The resultant cDNA was quantified by a Nanodrop 2000 Spectrophotometer (Thermo Fisher Scientific Inc., Agawam, MA). The qRT-PCR was performed on a 7500 Fast Real-Time PCR System (Applied Biosystems, Singapore) with PowerUP SYBR Green Master Mix (Applied Biosystems, Foster City, CA) using 200 ng of input cDNA. The reaction conditions were informed by manufacturer recommendations and optimized for the specific target locus. qRT-PCR primers were designed using the Primer3 software (Table S1; http:// frodo.wi.mit.edu). The gene Blon_0393, encoding a cysteinyl-tRNA synthetase was used as an endogenous control as previously (16, 41). Growth on lactose (2% wt/v) served as the reference condition for gene expression. Results were expressed as fold change relative to the reference. These experiments were conducted in triplicates and triplicate technical measurements were performed. Following DNase treatment, the absence of genomic DNA was confirmed using total RNA as template by qRT-PCR (i.e., endogenous control reaction).

### Statistical analyses

The relationships between asymptotic OD_600 nm_, growth rates and metabolites were characterized with principal components analysis (PCA) and hierarchical clustering with Ward's method and Euclidean distances using R (R.3.4.0). The outliers were determined according to their distance to the average within biological replicates were omitted to maintain at least biological triplicates. When no growth was observed in sugars, the values were assigned as “0” for PCA function(prcomp) analysis and PCA plots were drawn using ggbiplot in R. Growth kinetics, metabolite concentrations, fold change in gene expressions of cell culture for *B. infantis* ATCC 15697 were subjected to one-way analysis of variance (ANOVA) and Tukey's HSD test for multiple comparisons between carbohydrate source. The fold change in gene expression for *B. infantis*, growth kinetics, and metabolites between strains were analyzed with two-way ANOVA. The simple effects and main factor effects were determined with Tukey's HSD test for multiple comparisons of carbohydrate sources for the same strain and between strains for a defined carbohydrate source.

### Bioinformatic analysis of transcriptome data

Transcriptomic data (i.e., raw reads) of *B. infantis* ATCC 15697 while growing on lactose, LNT, and LNnT was retrieved from a previously performed RNA-seq study (26) publically deposited in the NCBI Gene Expression Omnibus database (http://www.ncbi.nlm.nih.gov/geo/) under the accession number GSE58773 (and personal communication with Danielle Lemay). This data was uploaded to the Massachusetts Green High Performance Computing Cluster (MGHPCC) that was used for all computational/statistical analyses unless specifically noted. The RNA-seq reads were aligned to the reference *B. longum* subsp. *infantis* ATCC 15697 genome (NC_011593.1). Coding regions of the ATCC 15697 genome were subjected to this analysis. Total and unique gene reads aligning to a specific genomic locus (i.e., locus tag), as well as calculated raw counts was obtained for differential expression analysis.

### Differential gene expression

In order to identify and quantify the magnitude of differentially expressed genes, the R package DESeq2 was used to analyze the raw count data (42). Genes with a mean count <200 was removed from analysis by pre-filtering. DESeq2 applies the Wald test for statistical analysis. Adjusted *p* ≤ 0.05 were defined as statistically significant.

### Anti-inflammation assay performed in a cell culture model

Caco-2 cells (ATCC HTB 37) lines were routinely cultured in High Glucose Dulbecco's Modified Eagle Medium (DMEM) (Corning, Manassas, VA) supplemented with NaHCO_3_ (Sigma-Aldrich, St. Louis, MO), 1% non-essential amino acids (Gibco, Dublin, Ireland), 100 U/ml penicillin-streptomycin (Gibco, Dublin, Ireland), 10% (v/v) fetal bovine serum (Seradigm VWR, Radnor, PA), and 7 mM HEPES (Gibco, Dublin, Ireland). Caco-2 cells were routinely grown in 20-cm Petri plates and subcultured at 80% confluence and maintained at 37°C in a humidified atmosphere of 5% (v/v) CO_2_ in air.

The cells were differentiated at passages 30–32 and collected by dissociation of a 90% confluent stock culture with 0.25% trypsin/EDTA (Gibco, Dublin, Ireland). For the inflammation assay, Caco-2 cells were seeded in 24-well plate at a concentration of 1–2 × 10^5^ cells/cm^2^ and were differentiated for 17 days with the medium changed every 2–3 days. Replicate supernatants collected from *B. infantis* ATCC 15697 growing on lactose, LNT, and LNnT were mixed in equal volume and added into the DMEM at the final concentration of 15% (v/v). One hundred microliter of acetic acid, lactic acid, and formic acid controls were mixed with DMEM. Subsequently, media was added to each well in triplicates and incubated at 37°C in a 5% CO_2_ atmosphere for 2 h. For negative and positive controls, triplicates were seeded with only DMEM. After incubation, 10 μl of 5 mg/ml lipopolysaccharide (LPS, Sigma-Aldrich, St. Louis, MO) in PBS was added into the wells and incubated for 24 h for treatments. LPS alone was used for negative control. PBS alone served as the carrier solution and a reference control. After incubation, the cells were detached from the plate surface by incubation with trypsin/EDTA, suspended in 500 μl of RNAlater solution and stored at −80°C until RNA extraction.

The relative gene expression of interleukin-8 (IL-8) linked with LPS-induced inflammation expression was quantified using qRT-PCR. Total RNA was extracted using the Ambion RNAqeous-Total RNA extraction kit (Invitrogen, Vilnius, Lithuania) according to the manufacturer's instructions. Cells suspended in lysis buffer were transferred to Lysing Matrix D tubes specific for eukaryotic cell and tissues culture (MP Biomedicals LLC, Solon, Ohio) and were subject to a speed of 5.5 m/sec for 30 sec twice using the FastPrep 24 bead beader (MP Biomedicals, Santa Ana, CA). The total RNA was eluted in 50 μl of EB solution and immediately subjected to DNase treatment with the Ambion Turbo DNA-free (Invitrogen, Vilnius, Lithuania) using 1 μl of DNase I for half-hour. Total RNA was converted to cDNA using the High Capacity cDNA Reverse Transcription Kit (Applied Biosystems, Carlsbad, CA) according to manufacturer's instructions. The resultant cDNA was quantified in a Nanodrop 2000 Spectrophotometer (Thermo Fisher Scientific Inc., Agawam, MA). The qRT-PCR analysis was performed using a 7500 Fast Real-Time PCR System (Applied Biosystems, Singapore) with PowerUP SYBR Green Master Mix (Applied Biosystems, Foster City, CA) using 200 ng of cDNA with primers GAPDH-F (5′-GTCGCTGTTGAAGTCAGAGG-3′) and GAPDH-R (5′-GAAACTGTGGCGTGATGG-3′) for endogenous control and primers IL-8-F (5′-GACCACACTGCGCCAACAC-3′) and IL-8-R (5′-CTTCTCCACAACCCTCTGCAC-3′) (43). The PCR cycling conditions were applied as recommended by the manufacturer and tailored specifically to the target genes. Additional markers of inflammation were tested using primers IL-10-F (5′-GGTTGCCAAGCCTTGTCTGA-3′), IL-10-R (5′-AGGGAGTTCACATGCGCCT-3′) (44), and TNF-α-F (5′-TCAACCTCCTCTCTGCCATC-3′), TNF-α-R (5′-CCAAAGTACACCTGCCCAGA-3′) (45).

## Results

### *B. longum* subsp. *infantis* exhibits divergent growth phenotypes during utilization of the milk oligosaccharides lacto-*N*-tetraose and lacto-*N*-neotetraose

**Figure 1:**
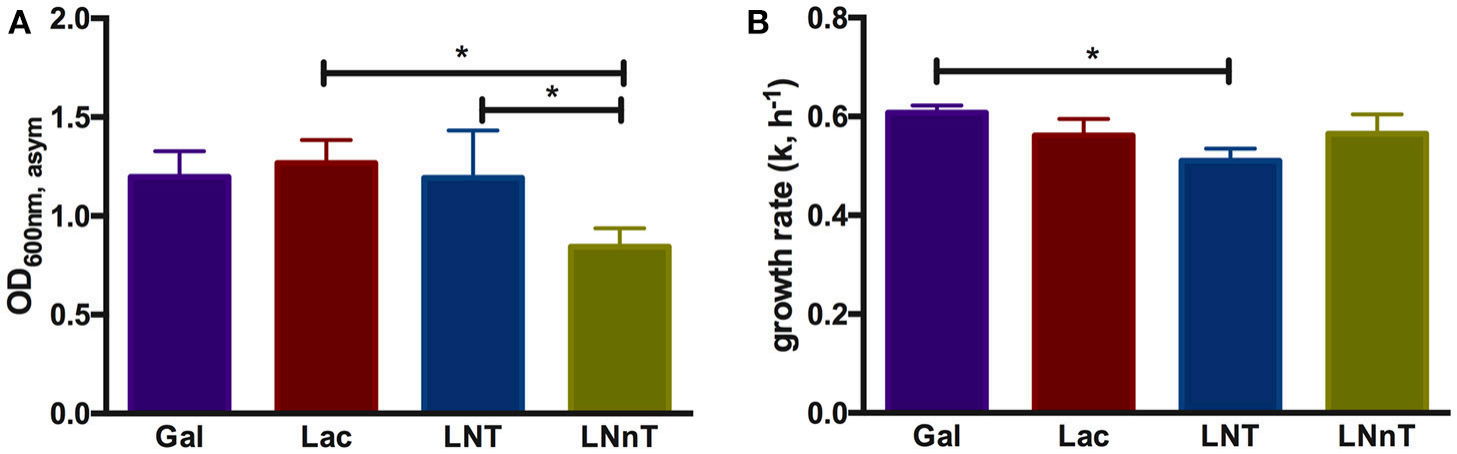
*B. longum* subsp. *infantis* ATCC 15697 growth kinetics while utilizing milk carbohydrates. The final asymptotic OD_600 nm_ **(A)** and growth rate (k, h^−1^) **(B)** of *B. infantis* ATCC15697 subsisting on mMRS medium containing 2% (wt/v) galactose (Gal), lactose (Lac), lacto-*N*-tetraose (LNT), or lacto-*N*-neotetraose (LNnT). The growth kinetics was calculated with Wolfram Mathematica 10.3. The data depicts mean ± *SD* of three independent experiments. The single asterisk (*) indicates the significant differences between carbohydrate utilizations evaluated by one-way ANOVA and Tukey's multiple comparison (*p* < 0.05).

In order to understand *B. infantis* metabolism of HMOs, the type strain ATCC 15697 was subjected to growth on purified HMO species and constituent mono- and di-saccharides. Accordingly, *B. infantis* ATCC 15697 grew vigorously on lactose (OD_600nm_, _asym_ = 1.27 ± 0.12, k = 0.56 ± 0.03 h^−1^) as well as galactose (OD_600nm_, _asym_ = 1.20 ± 0.13, k = 0.61 ± 0.02 h^−1^) (Figure 1). The HMO species LNT was utilized as a sole carbohydrate source to a similar extent (OD_600nm_, _asym_ = 1.19 ± 0.24, k = 0.51 ± 0.02 h^−1^) as these two constituent residues (Figure 1). Interestingly, the structural isomer LNnT promoted a more moderate growth profile (OD_600nm_, _asym_ = 0.85 ± 0.09, k = 0.57 ± 0.04 h^−1^) (*p* < 0.05) (Figure 1). The *B. infantis* type strain did not grow on the HMO constituents GlcNAc and fucose as a sole carbohydrate source. It is noteworthy that growth on glucose was inconsistent in terms of final OD_600 nm_; therefore, a comparison to the other carbohydrates was limited. The significant difference between LNT and LNnT utilization (*p* < 0.05) suggests that these HMOs are metabolized via differential mechanisms that vary in efficiency (Figure 1A). Differences in growth rates between the HMO species were not observed which indicates an equivalent preference for LNT and LNnT (Figure 1B). *B. infantis* ATCC 15697 exhibited similar growth rates for lactose, galactose, and LNnT (*p* > 0.05). Although growth efficiencies on galactose and LNT are similar (Figure 1A), *B. infantis* ATCC 15697 prefers galactose to LNT as indicated by growth rate (*p* < 0.05). In aggregate, the single structural difference between LNT and LNnT is directly linked to the efficiency by which *B. infantis* utilizes these HMO species. Previous studies indicate that ATCC 15697 grows on both LNT and LNnT to achieve a OD_600 nm_ > 0.8, however these studies did not report the specific asymptotic growth (i.e., efficiency) or growth rate (i.e., preference) when growing on these two HMO species (17, 26, 46, 47). Thus it is significant that ATCC 15697 exhibits clear differences in utilization between LNT and LNnT when accumulating biomass to OD_600 nm_ values >0.8.

### Metabolic endproducts are differentially secreted dependent on the milk oligosaccharide substrate lacto-*N*-tetraose or lacto-*N*-neotetraose

**Figure 2:**
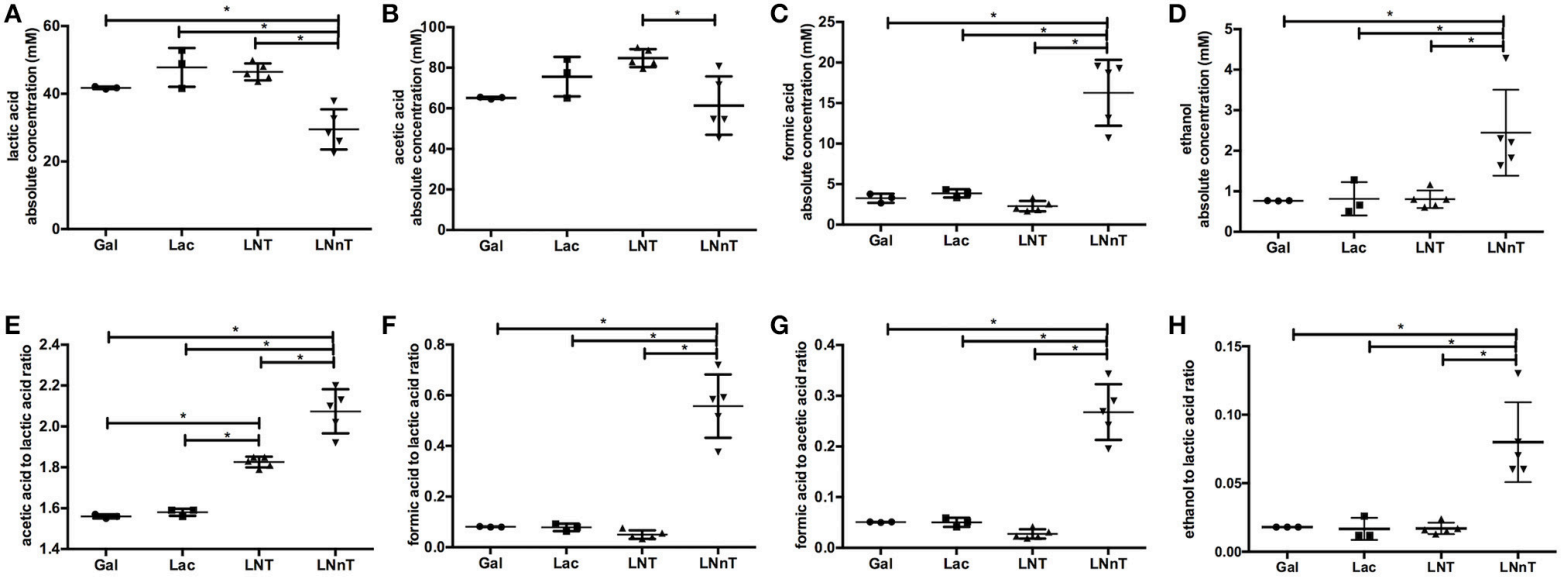
*B. longum* subsp. *infantis* ATCC 15697 fermentative endproducts while utilizing milk carbohydrates through the F6PPK pathway. Absolute concentrations of lactic acid **(A)**, acetic acid **(B)**, formic acid **(C)**, and ethanol **(D)**. In addition, acetic acid to lactic acid ratio **(E)**, formic acid to lactic acid ratio **(F)**, formic acid to acetic acid ratio **(G)**, and ethanol to lactic acid ratio **(H)**. All panels represent *B. infantis* ATCC15697 growing on mMRS medium containing 2% (wt/v) galactose (Gal), lactose (Lac), lacto-*N*-tetraose (LNT), or lacto-*N*-neotetraose (LNnT). Averages from independent biological replicates (triplicate or more) are shown with bars representing standard deviations of the means. The values for organic acid production are expressed in millimolar (mM) absolute concentration. A single asterisk (*) denotes significant differences between metabolite production evaluated by one-way ANOVA and Tukey's multiple comparison test (*p* < 0.05).

Fermentative endproducts were profiled to detail the metabolic consequences of HMO carbohydrate flux through the F6PPK pathway. During hexose fermentation, acetic acid and lactic acid are typically secreted in a theoretical ratio of 1.5. In contrast, formic acid production is not expected under most conditions tested to date (20, 47). The absolute concentrations of lactic acid, acetic acid, formic acid, ethanol, and the ratios between these metabolites are depicted in Figure 2. *B. infantis* ATCC 15697 produces similar concentrations of lactic acid while growing on galactose, lactose, and LNT (42.5 ± 5.7, 47.8 ± 5.7, and 46.5 ± 2.5 mM respectively) (*p* > 0.05, Figure 2A). A much lower lactic acid concentration, however, was secreted while utilizing LNnT (29.5 ± 5.9 mM) compared to the metabolism of other carbohydrates (*p* < 0.05). Interestingly, formic acid and ethanol concentrations were significantly higher while growing on LNnT (16.3 ± 4.1 and 2.5 ± 1.1 mM respectively). This is contrasted with the relatively smaller concentrations while growing on LNT, lactose, and galactose (*p* < 0.05, Figures 2C,D).

LNT metabolism resulted in the highest concentration of secreted acetic acid (84.8±4.4 mM, Figure 2B) and significantly differed from LNnT metabolism (*p* < 0.05). In general, fermentative endproduct concentrations are expected to be positively correlated with the final biomass (48), as more carbohydrates processed by the F6PPK pathway results in more organic acids secreted. Therefore, it is expected that lactic acid and acetic acid concentrations will be higher when greater biomass is achieved (47). However, acetic acid concentrations while utilizing galactose, lactose, and LNnT did not significantly differ from each other (*p* > 0.05). Overall, these data support the hypothesis that *B. infantis* deploys a different mechanism while utilizing LNnT than LNT.

### The ratio of secreted endproducts indicate an alternative pathway for lacto-*N*-neotetraose metabolism

**Figure 3:**
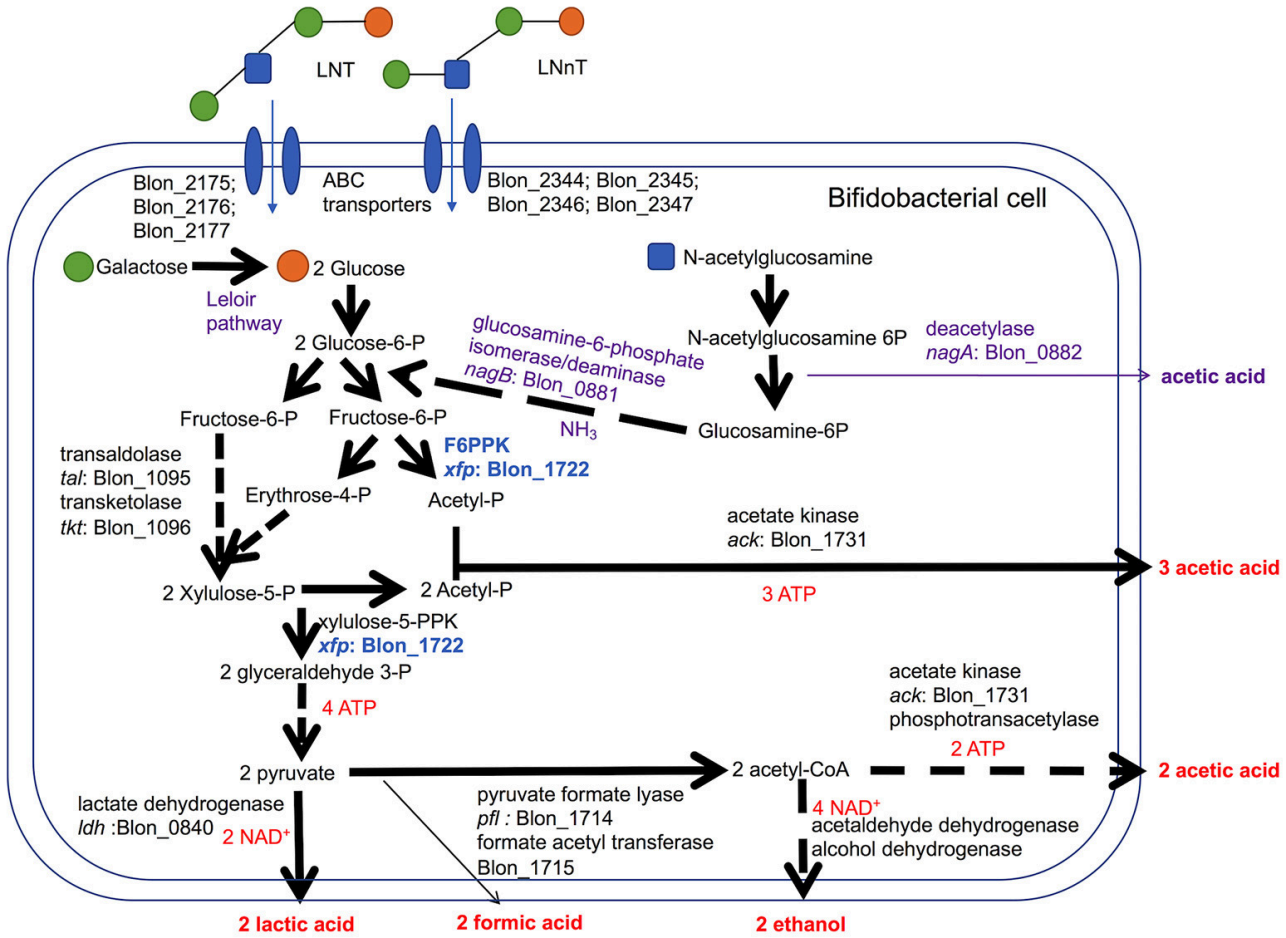
*Bifidobacterium longum* subsp. *infantis* metabolic pathways for utilization of lacto-*N*-tetraose (LNT) and lacto-*N*-neotetraose (LNnT) and their constituent monosaccharides. LNT and LNnT translocate through the cell membrane facilitated by ABC transporters. Intracellular glycosyl hydrolases process HMO into constituent monosaccharides to enter the central fermentative pathway. This pathway involves the characteristic fructose-6-phosphate phosphoketolase (F6PPK) activity denoted in blue. Genes encoding intracellular metabolic enzymes are depicted next to arrows according to their locus tag in the ATCC 15697 genome. Solid arrows are direct conversions with dashed arrows depicting the sequential actions of multiple enzymes. Predicted catabolic operations that feed into the F6PPK pathway and their corresponding products are denoted as purple. Stoichiometric coefficients of secreted metabolites, ATP, and NAD^+^ produced during metabolism are labeled in red. Experimental observations depicted in Figures 1, 2 including stoichiometry are incorporated.

Bifidobacteria, including *B. infantis*, catabolize 2 moles hexose to secrete 2 moles of lactic acid and 3 moles of acetic acid via the F6PPK pathway (Figure 3). This theoretical yield (i.e., acetate: lactate ratio of 1.5) was achieved during growth on galactose and lactose (1.56 ± 0.01 and 1.58 ± 0.01, respectively, Figure 2E). During HMO metabolism, LNT and LNnT utilization shifted the ratio toward greater acetic acid production (1.84 ± 0.01 and 2.08 ± 0.14, respectively, *p* < 0.05, Figure 2E). This is likely due to the deacetylation of the GlcNAc residue, at least in part. Notably this ratio significantly diverges between LNT and LNnT utilization with the latter experiencing a stronger shift (*p* < 0.05). If both HMO isomers increased the relative proportion of acetic acid via GlcNAc deacetylation, the higher ratio during LNnT metabolism is due to either decreased lactic acid production and/or increased acetic acid production from acetyl-CoA conversion.

LNnT metabolism was characterized by a significant increase in formic acid and ethanol production despite lower biomass. Accordingly, the ratio of formic acid to lactic acid was significantly higher during LNnT metabolism (*p* < 0.05, Figure 2F). Similarly, LNnT metabolism increased the formic acid to acetic acid ratio significantly (*p* < 0.05, Figure 2G). The theoretical formic acid to acetic acid ratio is 2:5 during more than 50% conversion of acetyl-CoA to acetic acid (25). This ratio was approached during LNnT metabolism (Figure 2G). This means, in part, that pyruvate is shunted toward acetyl-CoA resulting more formic acid and acetic acid production rather than lactic acid. Accordingly, ethanol to lactic acid ratio during LNnT fermentation differed significantly from LNT, as well as the other carbohydrates (*p* < 0.05, Figure 2H). The theoretical ratio is 1:1 when 50% of acetyl-coA is converted to ethanol. The higher ethanol to lactic acid ratio in LNnT indicates that ethanol production occurs for regenerating NAD^+^. The theoretical ratio has not been reached (~0.08), thus this explains that acetyl-CoA was mostly converted to acetic acid rather than ethanol to increase ATP production instead of NAD^+^ recycling. This indicates a clear metabolic shift toward these endproducts while subsisting on LNnT.

### Oligosaccharide transport gene expression remains similar regardless of lacto-*N*-tetraose and lacto-*N*-neotetraose substrates

**Figure 4:**
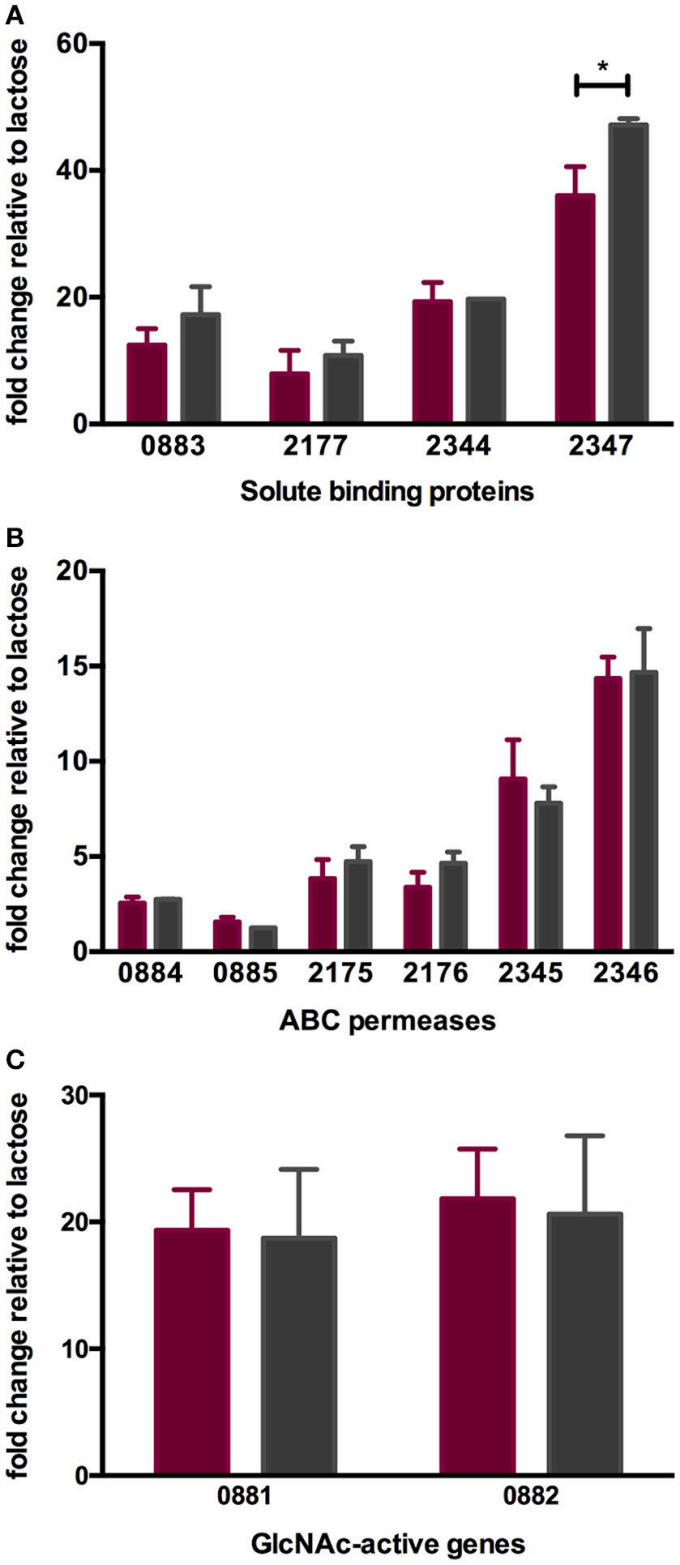
ATCC 15697 gene expression while subsisting on milk oligosaccharides as a sole carbon source. The x-axis represents gene locus tags of Family 1 solute binding proteins **(A)**, ABC permeases **(B)**, and GlcNAc-active genes **(C)**. The y-axis depicts the fold changes in gene expression relative to the control lactose as measured by qRT-PCR. LNT and LNnT are denoted in purple and gray. The error bars show standard deviations of three biological replicates except for LNnT. ^*^p < 0.05 evaluated by one-way ANOVA and Tukey's multiple comparison.

As with other bifidobacteria examined to date, *B. infantis* ATCC 15697 encodes several family 1 solute binding proteins (F1SBPs), ATP-binding domains, and permeases that assemble into ABC transporters with predicted affinity for oligosaccharides (10, 16, 49). The expression of four F1SBPs and their cognate ABC permeases during LNT and LNnT utilization was evaluated to test the hypothesis that transport contributes to the differential metabolic phenotypes (Figures 4A,B). These F1SBPs were previously identified to bind glycans that incorporate HMO moieties (16). Three F1SBPs (Blon_0883, Blon_2344 and Blon_2347) and four ABC permeases (Blon_2175, Blon_2176, Blon_2345 and Blon_2346) were induced more than 2-fold during the growth on LNT or LNnT as the sole carbon source relative to lactose (*p* < 0.05). Only Blon_2347 expression differed significantly between the metabolism of the two HMO species (*p* < 0.05, Figure 4A). Interestingly, both LNT and LNnT induced the F1SBP Blon_0883, although its adjacent permease proteins Blon_0884 and Blon_0885 were not induced (*p* > 0.05). Main effect analysis via two-way ANOVA indicates that Blon_2347 exhibits the strongest induction among the four F1SBP genes regardless of substrate (*p* < 0.05, Figure 4A). Among the permeases, it is notable that the highest relative expression occurred in transcription of Blon_2346, followed by Blon_2345 (*p* < 0.05, Figure 3B). These genes are located in a 40-kb catabolic cluster dedicated specifically to HMO metabolism (10). This indicates that *B. infantis* deploys HMO cluster transporters while utilizing both LNT and LNnT which differs significantly from its corresponding component, lactose. The differential metabolic phenotypes between LNT and LNnT are not linked to the expression of these transport genes, indicating that it is likely a function of intracellular catabolic operations.

### *B. infantis* upregulates *N*-acetylglucosamine metabolic genes while utilizing lacto-*N*-tetraose and lacto-*N*-neotetraose

During HMO hydrolysis, GlcNAc is liberated from the oligosaccharide and likely subjected to deamination and deacetylation before entering the F6PPK pathway (Figure 3). This is catalyzed by GlcNAc-6-phosphate deacetylase (*nagA*; Blon_0882, EC 3.5.1.25) and glucosamine-6-phosphate isomerase/deaminase (*nagB*; Blon_0881, EC 3.5.99.6). Both Blon_0881 and Blon_0882 exhibited significant upregulation while growing on LNT and LNnT relative to lactose (*p* < 0.05). Specifically, LNT induced fold changes of 19.34 ± 3.21 and 21.84 ± 3.90 of Blon_0881 and Blon_0882 respectively, whereas LNnT prompted a similar induction measured at 18.71 ± 5.43 and 20.61 ± 6.19 (Figure 4C). This upregulation is interpreted as consistent with GlcNAc catabolism providing evidence that deacetylation occurs during LNT and LNnT utilization. Significant differences in the expression of these GlcNAc genes were not detected between LNT and LNnT metabolism. This expression profile could reflect the growth rate similarity between LNT and LNnT as depicted in Figure 1B.

### The *B. infantis* transcriptome diverges during lacto-*N*-tetraose and lacto-*N*-neotetraose metabolism

The *B. infantis* ATCC 15697 transcriptome while utilizing HMOs was previously characterized by RNA-seq (26). Given the differential metabolism observed in the current study, specific pathways predicted to be relevant to LNT and LNnT utilization were examined in greater depth according to differential gene expression beyond normalized counts. Accordingly, raw reads were retrieved and subjected to differential expression analysis (i.e., 2-fold change) for HMO utilization cluster genes, galactose catabolic genes (i.e., Leloir pathway), GlcNAc-related genes, glycosyl hydrolases, and the F6PPK pathway as listed in Table S2.

**Figure 5:**
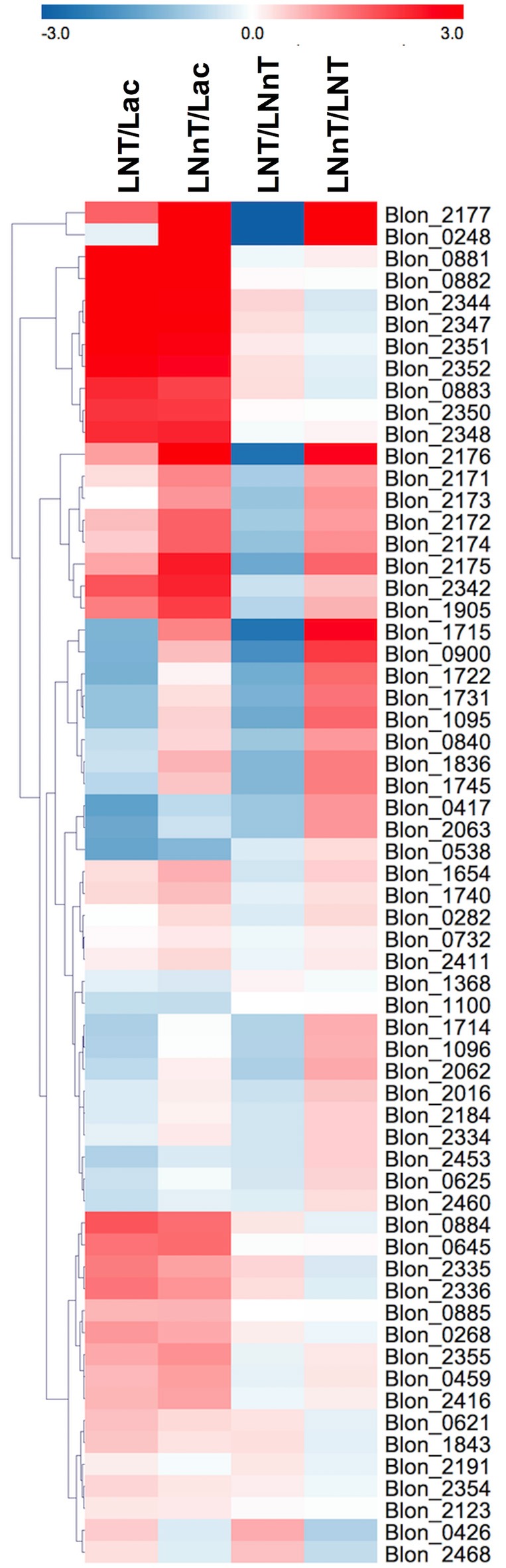
Relative gene expression within the global transcriptome depicted as log2-fold change. The log2-fold change gene expression from independent biological duplicates was performed from raw reads using the R package DESeq2. The 2-fold change expressions were subjected to hierarchical clustering using Euclidean distance and depicted as z-scores. Gene annotations and predicted functions are reported in Table S2.

Genes involved in galactose metabolism (Blon_2171, Blon_2172, and Blon_2174) and adjacent ABC transporters (Blon_2175, Blon_2176, and Blon_2177) are strongly upregulated by both LNT and LNnT compared to lactose (*p* < 0.05) (Figure 5). Moreover, LNnT prompted stronger induction of these genes relative to LNT (*p* < 0.05). Similarly, F1SBPs and permeases localized to the HMO utilization cluster (i.e., Blon_2344-2352) were upregulated by both LNT and LNnT relative to the lactose control (*p* < 0.05). In addition, Blon_2344, Blon_2347, and Blon_2352 were significantly upregulated during LNT fermentation when compared to LNnT (*p* < 0.05).

The GlcNAc utilization genes Blon_0881 (*nagB*) and Blon_0882 (*nagA*) were significantly upregulated while *B. infantis* utilizes both LNT and LNnT relative to lactose (*p* < 0.05, Figure 5). However, there is no significant difference between LNT and LNnT metabolism (*p* > 0.05). This is consistent with the qRT-PCR gene expression analysis.

The key enzyme fructose-6-phosphate phosphoketolase (*xfp*, Blon_1722) has been postulated to be highly expressed regardless of carbohydrate substrate (26). Interestingly, F6PPK pathway genes are downregulated by LNT relative to lactose and LNnT (*p* < 0.05, Figure 5). In addition, LNnT showed strong upregulation of those genes compared to lactose (*p* < 0.05), except for Blon_1722, Blon_1096, and Blon_1368 that did not significantly differ (*p* > 0.05). This is interesting as the biomass and growth rate achieved with LNT or lactose did not significantly differ (*p* > 0.05, Figure 1). Despite the potential for greater flux through the central fermentative pathway as per the transcriptome, LNnT prompted less biomass production (*p* < 0.05). Furthermore, lactate dehydrogenase (*ldh;* Blon_0840, EC 1.1.1.37), converts pyruvate to lactate to recycle cofactors and was significantly induced by LNnT relative to lactose and LNT (*p* < 0.05). The relationship between *ldh* expression and LNnT inducing lower lactic acid concentrations is unclear. This is potentially indicative of variation between the physiological state of the cells during sample collection (i.e., mid-exponential or stationary phase). Accordingly, high levels of lactic acid was observed at the beginning of fermentation of oligofructose by *B. animalis* and replaced by formic acid at later stages (50).

Interestingly, and potentially underlying differential metabolism, acetate kinase (*ack*; Blon_1731, EC 2.7.2.1) was more strongly upregulated while consuming LNnT relative to lactose and LNT (*p* < 0.05). Acetate kinase catalyzes substrate-level phosphorylation in the F6PPK pathway that is both involved in conversion of phosphoketolase to acetyl-P and conversion of acetyl-coA to acetate, and thus reflects relatively higher acetic acid secretion during LNnT fermentation.

As LNnT utilization is characterized by increased formic acid production, putative genes involved in this pathway were interrogated. It is noteworthy that this metabolic process is incompletely characterized in the bifidobacteria. Formate acetyl transferase (Blon_1715, EC 2.3.1.54) and pyruvate formate lyase (Blon_1714, EC 1.97.1.4) potentially converts pyruvate to acetyl-coA and produces formic acid. Accordingly, Blon_1715 is highly expressed during LNnT utilization relative to lactose and LNT (*p* < 0.05). Conversely, LNT metabolism is not characterized by increased formic acid production and prompted a downregulation of both Blon_1714 and Blon_1715 relative to lactose and LNnT (*p* < 0.05). Although F6PPK genes were upregulated by LNnT relative to lactose, the strongest change was observed in formate acetyl transferase (2-fold change = 1.20). Again, this is consistent with increased formic acid production during LNnT metabolism to provide evidence that differential phenotypes exhibited between LNnT and LNT is regulated at the gene expression level, at least in part.

Twenty five key glycosyl hydrolases (GHs) were selected for further analysis (Figure 5) (10, 17, 51). A total of 13 loci significantly differ between LNT and LNnT metabolism (*p* < 0.05). Among β-galactosidases, Blon_2016 (GH family 42), and Blon_2334 (GH family 2) were downregulated during LNT utilization relative to lactose and LNnT (*p* < 0.05). This is interesting because Blon_2016 was shown to have specificity to type I HMOs such as LNT (52) and Blon_2334 was shown to be constitutively expressed in the utilization of pooled HMOs and other complex oligosaccharides (26, 51). β-glucosidase Blon_1905 was significantly upregulated by both LNT and LNnT compared to lactose (*p* < 0.05) with expression during LNnT growth significantly higher than LNT (*p* < 0.05).

Salient to general HMO metabolism, an α-L-fucosidase Blon_0248 (GH family 29) was significantly upregulated by LNnT compared to lactose and LNT (*p* < 0.05). Interestingly, another GH 29 α-L-fucosidase (Blon_0426) is strongly upregulated by LNT whereas it is downregulated by LNnT (*p* < 0.05). Other fucosidases Blon_2335 and Blon_2336 were strongly upregulated by LNT rather than LNnT (*p* < 0.05). The glycosyl hydrolases, Blon_0625 and Blon_2460 were downregulated in both HMO species compared to lactose, with a significantly stronger downregulation observed in LNnT than LNT (*p* < 0.05). Blon_2468, an endo-β-*N*-acetylglucosaminidase, which generally releases *N*-glycans from human milk glycoproteins was upregulated by LNT while it was downregulated by LNnT (*p* < 0.05).

### *B. longum* subsp. *infantis* exhibits growth phenotype variance while utilizing lacto-*N*-tetraose and lacto-*N*-neotetraose in a strain-dependent manner

In order to evaluate potential phenotypic variation within *B. infantis*, three strains in addition to ATCC 15697 were subjected to growth on glucose, galactose, lactose, LNT, and LNnT as a sole carbon source (Table S3). Both *B. infantis* UMA299 and UMA300 utilized GlcNAc as a sole carbohydrate substrate in contrast to ATCC 15697. None of the *B. infantis* strains tested utilized fucose as a sole fermentative substrate. UMA299 exhibited lower growth on LNT (OD_600nm_, _asym_ = 0.69 ± 0.09, k = 0.57 ± 0.05 h^−1^) and LNnT (OD_600nm_, _asym_ = 0.71 ± 0.06, k = 0.68 ± 0.06 h^−1^) compared to constituent carbohydrate residues within HMOs (*p* < 0.05, Table S3). This is significant, as UMA299 does not utilize pooled HMOs efficiently in contrast to other *B. infantis* strains (31). This is likely due to the absence of two F1SBP transporter genes (Blon_2344 and Blon_2351) in its HMO catabolic cluster (30). Interestingly, and despite limited growth, UMA299 exhibited a higher preference for LNnT with significantly lower growth rate on LNT (*p* < 0.05). In addition, UMA299 exhibited a OD_600nm_, _asym_ of 0.42 ± 0.09 on soluble GlcNAc with a growth rate of 0.16 ± 0.01 h^−1^ which is significantly lower compared to other carbohydrates tested (*p* < 0.05).

*B. infantis* UMA300 utilized LNnT efficiently (OD_600nm_, _asym_ = 1.30 ± 0.12, k = 0.51±0.05 h^−1^) which is to the same extent as LNT (OD_600nm_, _asym_ = 0.99 ± 0.20, k = 0.71 ± 0.05 h^−1^). The strain utilized galactose to similar cellular concentrations as LNT and LNnT and inefficiently utilizes lactose and GlcNAc (Table S3). In terms of growth rate (k), UMA300 experienced the highest rate on LNT and galactose with a significantly lower growth rate on LNnT (*p* < 0.05). This provides additional evidence that the two HMO species are utilized by divergent mechanisms by *B. infantis* strains. Despite achieving highest biomass concentrations on LNnT, UMA300 preference as determined by growth rate did not vary appreciably between LNnT and lactose (*p* > 0.05). UMA300 low growth rate on GlcNAc suggests that this aminosugar is not preferred relative to the other carbohydrates tested (*p* < 0.05).

In contrast to UMA300, *B. infantis* UMA301 exhibited vigorous growth on lactose (OD_600nm_, _asym_ = 1.29 ± 0.05, k = 0.56±0.05 h^−1^), followed by galactose (OD_600nm_, _asym_ = 1.22 ± 0.05, k = 0.63 ± 0.06 h^−1^), and LNT (OD_600nm_, _asym_ = 1.01 ± 0.17, k = 0.44 ± 0.06 h^−1^) all of which does not differ significantly (*p* > 0.05, Table S3). UMA301, however, achieved significantly lower biomass concentrations on LNnT (OD_600nm_, _asym_ = 0.86 ± 0.04, k = 0.40 ± 0.01 h^−1^) concomitant with a lower growth rate. This suggests a clear preference for LNT rather than LNnT between these two HMO tetrasaccharides. As with ATCC 15697, UMA301 does not utilize GlcNAc as a sole carbohydrate source.

Consistent with the growth, ATCC 15697, UMA299, and UMA301 consumed galactose and lactose >40%, whereas UMA300 consumed lactose at 25% (*p* < 0.05, Figure S1A). Similarly, UMA299 consumed GlcNAc to a greater extent than UMA300.

**Figure 6:**
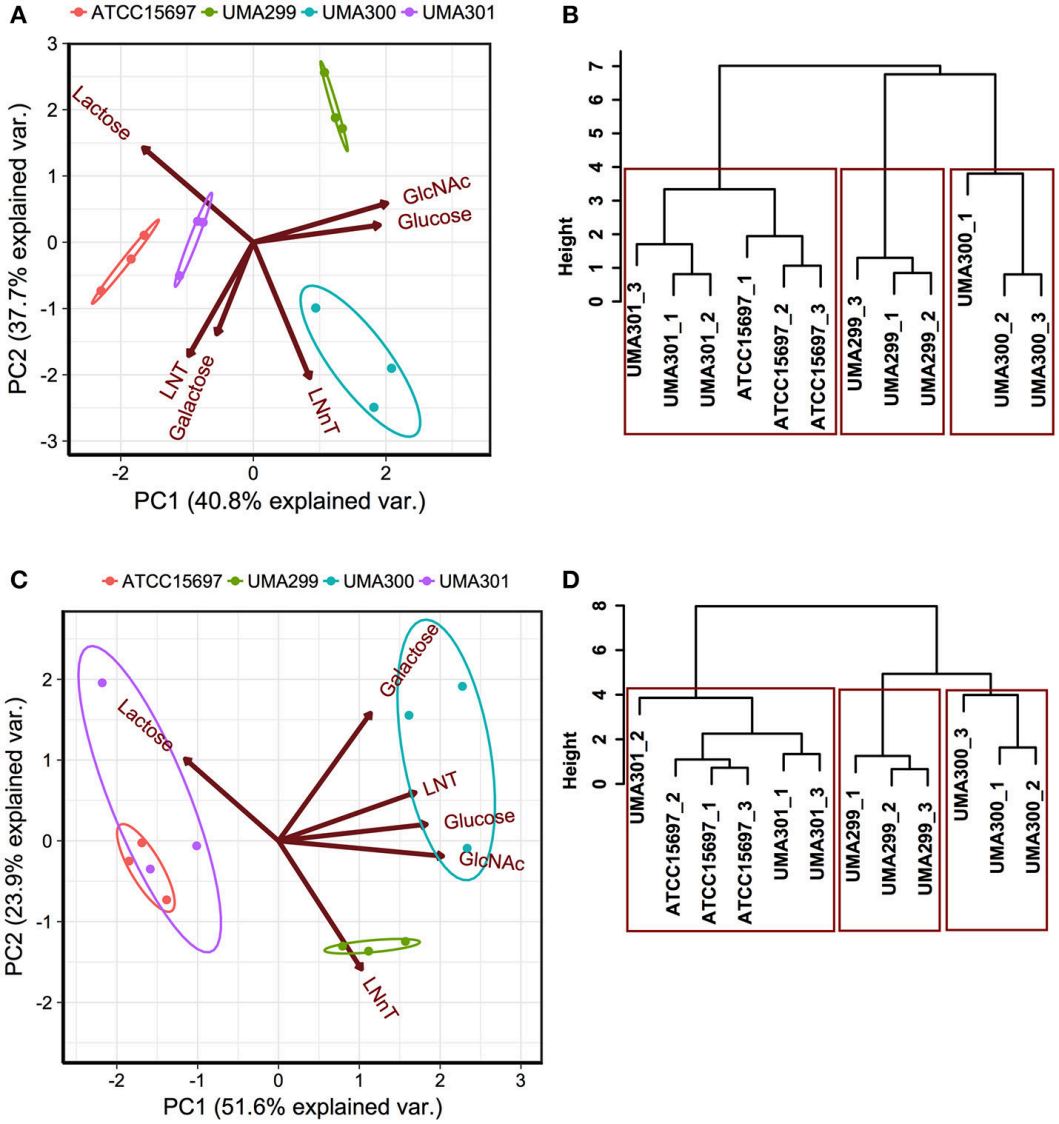
Strain dependent variation of human milk oligosaccharide utilization. Panels **(A,C)** depict the 2D-principal component analysis (PCA) performed on the final asymptotic OD_600_ and the growth rate (k, h^−1^) respectively. The arrows in PCA plot represent the correlation of variables with the principal components (PC1 and PC2). Points represent the scores of each component grouped as biological replicates. The ellipses encompassing each strain capture 68% of the normal probability of the scores within corresponding strains. Panels **(B,D)** show the hierarchical clustering dendrogram of strains on the final asymptotic OD_600 nm_ and the growth rate (k, h^−1^) using ward method and Euclidean distance. The y-axis measures closeness of either individual strains or clusters. The boxes show the 95% of clustering and closeness of the strains.

**Figure 7:**
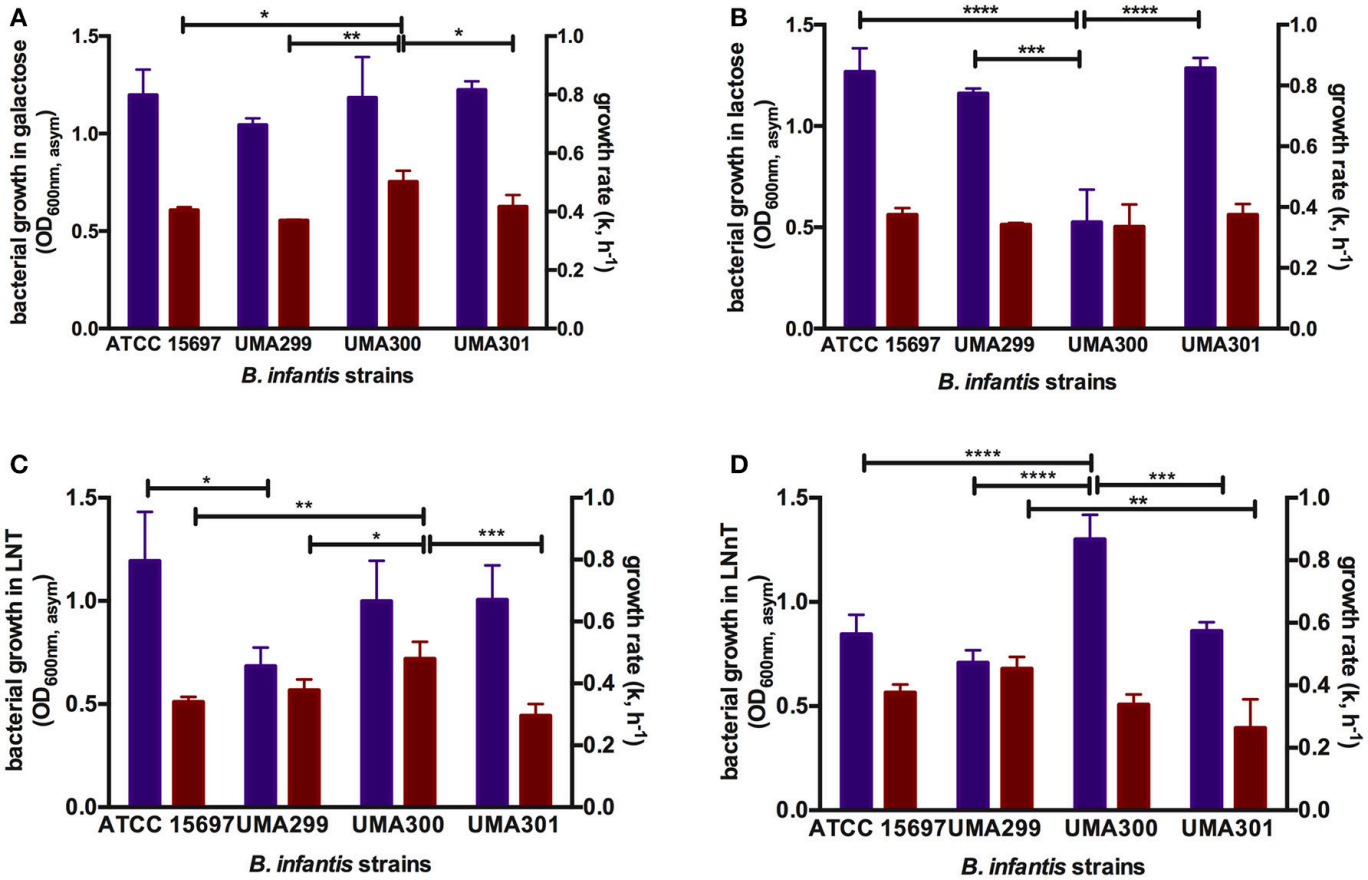
Growth kinetics of *B. longum* subsp. *infantis* strains subsisting on milk carbohydrates. The final asymptotic OD_600 nm_ and growth rate (k, h^−1^) of *B. infantis* strains while growing on mMRS medium containing 2% (wt/v) galactose **(A)**, lactose **(B)**, lacto-*N*-tetraose (LNT) **(C)**, and lacto-*N*-neotetraose (LNnT) **(D)**. The growth kinetics was calculated with Wolfram Mathematica 10.3 and represents the mean ± *SD* of three independent experiments. Purple and red bars indicate bacterial growth and growth rate respectively. The asterisks indicate the significant differences between strains evaluated by one-way ANOVA and Tukey's multiple comparison. ^*^p < 0.05, ^**^p < 0.005, ^***^p < 0.0005, and ^****^p < 0.0001. The growth kinetics for glucose and GlcNAc were not included as not all strains consume these monosaccharides.

In order to determine phenotypic variation as a function of carbohydrate source and *B. infantis* strain, principal component analysis (PCA) and hierarchical clustering was performed. This analysis incorporated both final asymptotic OD_600 nm_ and growth rate data for strains growing on individual substrates (Figure 6). The first principal component (PC1) explains 40.8% variation in OD_600nm_, _asym_ values, whereas PC2 captures 37.7% variation (Figure 6A). The scores of each component grouped as strains clustered closely (i.e., within the normal probability). This indicates that the growths are consistent among biological replicates regardless of fermentative substrate. Arrows oriented toward the same direction denote that growth on a particular carbohydrate is correlated with that PC component with the angle between arrows indicating a similar response profile. LNT and galactose utilization vectors emanated toward similar directions that is negatively correlated with both PCs (Figure 6A). These two vectors aligned along PC1 where ATCC 15697 and UMA301 growths clustered. UMA300 growth, however, aligned positively with PC1 in the same direction as the LNnT utilization vector. This indicates that UMA300 utilized LNnT well relative to other carbohydrates and strains. This is consistent with strain-dependent utilization, as UMA300 had a higher final OD_600nm_, _asym_ during LNnT utilization compared to other strains (Figure 7D). Interestingly, the LNnT and lactose utilization vectors were oriented in opposing directions suggesting utilization differences between them. GlcNAc and glucose utilization had a similar alignment and positively correlated with both components where UMA299 clustered, which is consistent with its limited ability to utilize HMO efficiently.

Hierarchical clustering was employed to determine quantitative similarities between strains. The height between the lines indicates the distances between the strains and their biological replicates (Figure 6B). The PCA and hierarchical clustering were concordant, and indicated that ATCC 15697 and UMA301 clustered together with the height of <4 (Figure 6B). Interestingly, this reflects the phylogenic relationship between ATCC 15697 and UMA301 (30).

In addition to final biomass achieved, PCA of growth rates observed on multiple substrates resolved onto PC1 to encompass 51.6% variability for each strain and positively correlated with all carbohydrates except lactose (Figure 6C). This is interesting as *Bifidobacterium* strains were previously determined to possess a preference for lactose over glucose (41), with lactose often used as a positive control and propagation of bifidobacterial strains. ATCC 15697 and UMA301 co-clustered and negatively correlated with PC1. These two strains exhibited similar utilization rates for all substrates tested. Interestingly, UMA300 growth rates on galactose, LNT, glucose, and GlcNAc were similar and distinctly clustered from other strains (Figure 6C) with significantly higher values on these carbohydrate sources compared to other strains (*p* < 0.05, Figure 7). Hierarchical clustering using the Ward method validated the PCA (Figure 6D). Hierarchical clustering of the growth rate is also consistent with the same analysis of the final biomass (OD_600nm_, _asym_), which yielded a similar distance topology. Accounting for the empirical evidence in aggregate, *B. infantis* utilization of LNT and LNnT diverges in a strain-dependent manner.

### *B. longum* subsp. *infantis* strains differentially metabolize lacto-*N*-tetraose and lacto-*N*-neotetraose

**Figure 8:**
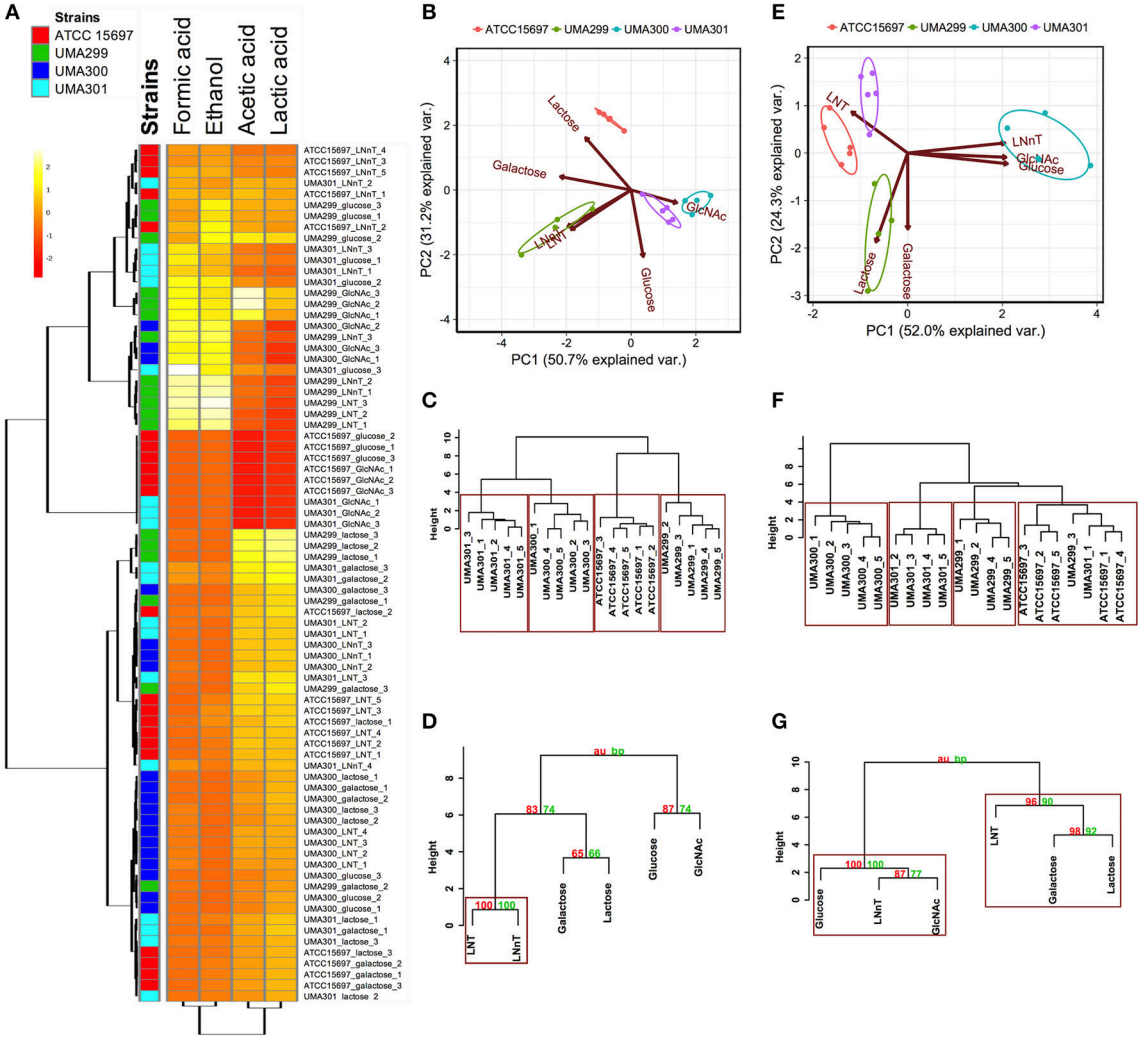
Analysis of *B. longum* subsp *infantis* strains secreted fermentative endproducts while utilizing milk carbohydrates. Panel **(A)** shows metabolites secreted by *B. infantis* strains for each carbohydrate clustered by Euclidean distance calculated with MetaboAnalyst 3.0. The scaling was performed by mean-centering and dividing by the standard deviation of each metabolite. The red denotes lower concentrations of the metabolite with yellow approaching higher concentrations. Panels **(B,E)** display the 2D-principal component analysis (PCA) plot depicting acetic acid to lactic acid ratios and acetic acid to lactic acid to formic acid ratios respectively. The arrows in PCA plot represent the correlation of the variables with the principal components (PC1 and PC2). Points represent the scores of each component grouped as biological replicates. The ellipses for each strain incorporate 68% of the normal probability of the scores for corresponding strains. Panels **(C,F)** are hierarchical clustering dendrogram of strains according to acetic acid to lactic acid ratios and acetic acid to lactic acid to formic acid ratios using the Ward method and Euclidean distance. The y-axis measures the closeness of either individual strains or their calculated clusters. Panels **(D,G)** represent hierarchical clustering based on *p*-values calculated with multiscale bootstrapping between acetic acid to lactic acid ratio and acetic acid to lactic acid to formic acid ratio. The y-axis measures the closeness of either individual substrates or clusters according to two metrics: Approximately Unbiased *p*-value (AU in red) and Bootstrap Probability value (BP in blue). Clusters exhibiting AU values >95% are highlighted by rectangles.

A comparative analysis of the fermentative endproducts lactic acid, acetic acid, formic acid, and ethanol was conducted on the panel of *B. infantis* strains. These endproducts are secreted as a result of carbon flux through the F6PPK pathway with data reported as a heatmap with hierarchical clustering analysis (Figure 8A). As expected, acetic acid and lactic acid production clustered more closely with each other than the ethanol and formic acid production as these two endproducts are secreted regardless of substrate (Figure 8A).

The metabolic profiling of ATCC 15697 and UMA301 while fermenting galactose, lactose, and LNT closely clustered together with high acetic acid and lactic acid and less formic acid and ethanol produced (Figure 8A). Interestingly, UMA299 produced higher concentrations of formic acid and ethanol when utilizing GlcNAc, glucose, LNT, and LNnT relative to other carbohydrates (Figure 8A). This indicates that UMA299 utilizes LNT via a different metabolic trajectory relative than other strains. This is consistent with its final biomass and general phenotype as an atypical HMO consumer (Figure 7C).

ATCC 15697, UMA301, and UMA299 LNnT metabolism was linked to higher formic acid and ethanol concentrations. UMA300, in contrast, exhibited a significantly different metabolic profile that is closely linked to LNT with less formic acid and ethanol production than the other strains (Figure 8A). This did not differ appreciably from galactose, lactose, and glucose metabolism. This suggests that UMA300, unlike other strains, preferentially converts pyruvate to lactic acid rather than acetyl-CoA regardless of the substrate with the exception of GlcNAc.

The carbon recovery observed in secreted metabolites after utilization of mono- and di-saccharides are depicted in Figure S1B. UMA299 recovered almost 100% carbon for all sugars except galactose. All strains recovered <100% carbon while fermenting galactose. ATCC 15697, UMA299, and UMA301 achieved almost 100% carbon recovery while utilizing lactose. Interestingly, UMA300 exhibited 138% of carbon recovery as determined by secreted metabolite following growth on lactose. This was significantly higher than ATCC 15697 and UMA301 (*p* < 0.05). This might be due to hydrolysis of the remaining lactose into glucose and galactose in the post- fermentation medium with UMA300 potentially exhibiting a preference for utilizing monosaccharides over lactose.

**Figure 9:**
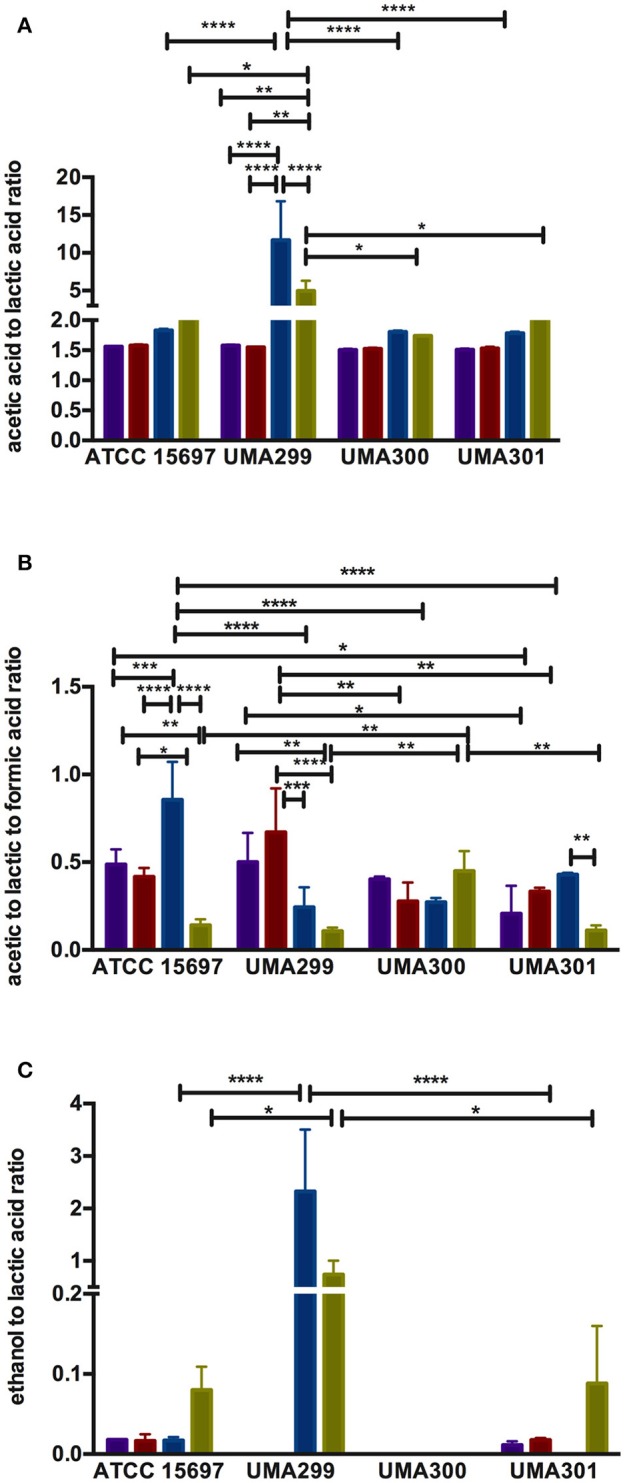
Endproduct ratios of *B. longum* subsp. *infantis* strain fermentative endproducts while utilizing the milk carbohydrates. Acetic acid to lactic acid ratio **(A)**, acetic acid to lactic acid to formic acid ratio **(B)**, and ethanol to lactic acid ratio **(C)**. Colors indicate the following carbohydrate substrates: Purple, galactose; red, lactose; dark blue, lacto-*N*-tetraose; green, lacto-*N*-neotetraose. Averages from independent biological replicates (at least triplicate) are shown with bars representing standard deviation from the mean. The asterisks indicate the significant differences evaluated by two-way ANOVA and Tukey's multiple comparison. ^*^p < 0.05, ^**^p < 0.005, ^***^p < 0.0005, and ^****^p < 0.0001.

The acetic acid:lactic acid ratios secreted after *B. infantis* strains fermented substrates were compared (Figures 8B–D). Accordingly, PC1 and PC2 encompass 50.7 and 31.2% of the variation for these ratios (Figure 8B). UMA300 and UMA301 displayed a positive correlation with PC1 whereas ATCC 15697 and UMA299 were negatively correlated. This is due to higher ratio values of strains on a particular carbohydrate with each strain clustering distinctly on PC2. The hierarchical clustering of strains was consistent with the PCA (Figure 8C). This is because UMA299 showed a significantly higher ratio in both LNT and LNnT metabolism relative to other strains (*p* < 0.05, Figure 9A). In addition, the direction of the LNT and LNnT vectors are negatively oriented with both components with a highly similar magnitude and direction in the PCA (Figure 8B). This explains the close clustering of LNT and LNnT based on Euclidean distance (< 0.95) whereas the HMO carbohydrate constituents segregated away from both HMO species (Figure 8D). The agglomerative clustering of strains revealed no differences for LNT and LNnT. This is potentially due to GlcNAc deacetylation increasing acetic acid production during HMO utilization.

Formic acid production underlies divergent mechanisms for LNT and LNnT metabolism (Figures 8E–G). Accordingly, the PCA performed on the acetic acid:lactic acid:formic acid ratios clearly indicates that LNT and LNnT fermentation proceeds via distinctive metabolic routes as these vectors are oriented in opposing directions along PC1 (captures 52.0% variation) (Figure 8E). This suggests that *B. infantis* strains shunt LNnT catabolism toward conversion of pyruvate to acetyl-CoA rather than lactic acid to subsequently secrete formic acid.

UMA300 was positively positioned along PC1 whereas ATCC 15697 and UMA301 were primarily explained by PC2. Interestingly, ATCC 15697 and UMA301 do not cluster together as depicted in Figure 8F despite exhibiting phenotypic and phylogenetic similarities. However, if the significance of height in the hierarchical clustering is increased, they cluster together, and only UMA300 stands alone. This is due to UMA300 not significantly secreting formic acid except during GlcNAc fermentation (Figure 8A). *B. infantis* ATCC 15697 and UMA301 produced significantly more formic acid during LNnT fermentation than LNT that resulted in a decrease of the ratio (*p* < 0.05, Figure 9B).

The ethanol to lactic acid ratio was dependent on the carbohydrate source and strain (Figure 9C). UMA300 did not produce ethanol regardless of the substrate whereas UMA299 exhibited a higher ratio for both LNT and LNnT relative to other strains (*p* < 0.05). Interestingly, UMA301 had limited ethanol production during LNT fermentation while the ratio increased in LNnT utilization. This means that all strains except UMA300 utilize LNnT along a similar metabolic route. Thus LNnT utilization by ATCC 15697 and UMA301 might involve NAD^+^ regeneration through ethanol production whereas recycling NAD^+^ during LNT utilization most likely occurs as pyruvate is converted to lactic acid.

### Human milk carbohydrate utilization mitigates lipopolysaccharide-induced IL-8 expression in caco-2 epithelial cells

**Figure 10:**
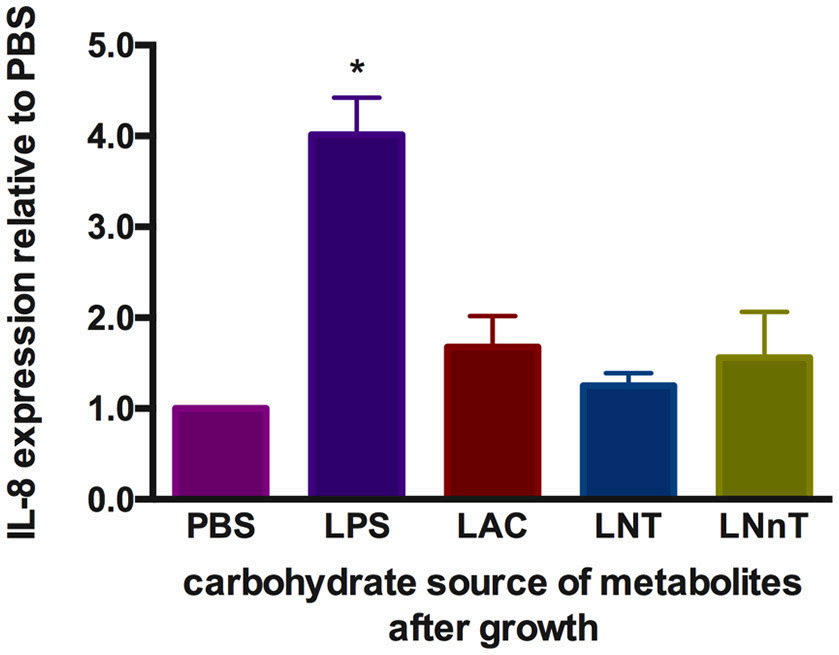
Gene expression of inflammatory marker Interleukin-8 in Caco-2 epithelial cells exposed to spent media following milk oligosaccharide fermentation. The y-axis represents the fold change in IL-8 expression relative to phosphate buffer solution (PBS). The x-axis depicts the sources of *B. infantis* metabolites which are used to treat Caco-2 cells after lipopolysaccharides (LPS) induction. The error bars show standard deviations of biological duplicates, each measured with three technical replicates. The single asterisks (*) indicate the significant differences evaluated by one-way ANOVA and Tukey's multiple comparison (*p* < 0.05).

It is known that metabolism of specific carbohydrates may influence bifidobacterial interactions with intestinal epithelia under certain conditions. Pooled HMO-grown bifidobacteria reduce inflammatory markers compared to glucose or lactose-grown bifidobacteria (53, 54). These previous studies examined adhesive properties of bifidobacteria and bacterial translocation in exponential growth instead of cell-free supernatants collected at stationary phase. Differential metabolism of LNT and LNnT inspired the hypothesis that host-microbial interactions may be influenced in a milk oligosaccharide-dependent manner. In order to address this, metabolites present in spent media subsequent to *B. infantis* growth on lactose, LNT, and LNnT were evaluated for their ability to mitigate inflammation. Specifically, it was hypothesized that higher acetic acid and formic acid concentrations secreted from LNnT metabolism will differentially influence inflammation. Gene expression of the cytokine marker of inflammation IL-8 was measured in Caco-2 cells following lipopolysaccharide-induced inflammation (Figure 10). Accordingly, spent media from all three fermentations significantly reduced IL-8 expression compared with the negative control (*p* < 0.05). However, there was no significant difference between the three milk carbohydrates. In addition, the markers of inflammation IL-10 and TNF-α were assayed and yielded inconsistent and thus inconclusive results. Although *B. infantis* metabolism of these human milk carbohydrates protects against inflammation, it is unclear to what extent that *B. infantis* alone is responsible for the anti-inflammatory effect. In addition, purified acetic acid, lactic acid, and formic acid were tested, however, the results were inconclusive due to variation between biological replicates.

## Discussion

*Bifidobacterium longum* subsp. *infantis* evolved to utilize glycans secreted in human milk to generate ATP as well as provide substrates for anabolic processes. Accordingly, its genome incorporates a 40-kb locus dedicated to human milk oligosaccharide utilization that is conserved in all *B. infantis* strains isolated to date (10, 30). The activities encoded by the HMO gene cluster allocate degradation products to be further metabolized prior to entering the F6PPK pathway, the characteristic fermentative pathway unique to the *Bifidobacterium* genus (20). HMOs evade digestion during gastrointestinal tract transit and thus are available to *B. infantis* to translocate intracellularly (16, 29). The F6PPK pathway terminates invariably in the extracellular secretion of acetic acid and lactic acid, with formic acid and ethanol generated to a lesser extent under specific conditions (20–23). The potential for *B. infantis* to differentially metabolize purified HMO species has not been fully tested. The HMO tetrasaccharides LNT and LNnT differ by a β1-3 and β1-4 linkage between galactose and *N*-acetylglucosamine at the non-reducing terminus respectively. Accounting for this structural variance, we hypothesized that LNT and LNnT are differentially metabolized after initiating distinct transcriptomic cascades to process these HMOs.

Previous research conducted on *B. infantis* provided the preliminary observations to generate this hypothesis (17, 26). In this current study, the model HMO-consuming strain *B. infantis* ATCC 15697 exhibits higher growth efficiency (i.e., asymptotic final OD) while metabolizing LNT rather than LNnT. This occurred in the absence of a preference for LNT over LNnT extrapolated from their similar growth rates. Thus ATCC 15697 may experience enhanced fitness when encountering LNT in the infant gut, although this remains to be tested in an *in vivo* system. Moreover, ATCC 15697 diverges in the metabolic fate of carbons during LNT or LNnT utilization. The ratio of secreted acetic acid to lactic acid (AA:LA) is considerably higher for LNT and LNnT than other carbohydrates. Importantly, LNnT promotes a significantly higher AA:LA ratio relative to LNT. The deacetylation of GlcNAc via deacetylase activity (EC 3.5.1.25, Figure 3) likely contributes to the increase in relative concentrations of acetic acid during LNT metabolism.

Of particular importance is that LNnT metabolism significantly increases formic acid production. This is not observed during LNT metabolism and constitutes a major metabolic shift solely attributable to the isomeric composition of LNnT. Thus the AA:LA ratio increases during LNnT fermentation likely due to GlcNAc deacetylation and a simultaneous decrease in lactic acid in shunting pyruvate toward formic acid production. The conversion of pyruvate to acetyl-CoA and subsequently to acetic acid during LNnT utilization results in formic acid and ethanol production. Modulation of acetic acid production produces higher levels of ATP during LNnT fermentation. This is consistent with the relative inefficiency LNnT is utilized for biomass as limited ATP restricts cellular growth. Bifidobacteria are known to increase formic acid secretion during inefficient metabolism of unfavorable substrates (25, 50, 55–57). It is noteworthy that previous studies observed cellular growth considerably lower than the accumulated biomass generated on LNnT in the present study.

In addition, increased ethanol production during LNnT metabolism recycles NAD^+^ following reduction of acetyl-coA. Since formic acid is generated at the expense of lactic acid, recovering NAD^+^ is critical as lactic acid production recycles cofactors and does not yield ATP (23). On a molar basis the 2-carbon pathway terminating in ethanol recoups double the amount of NAD^+^ than the 3-carbon arm (i.e., pyruvate to lactic acid). Whereas it is clear that LNnT shifts metabolism toward formic acid and ethanol production, the molecular mechanisms underlying these alternative pathways remains incompletely understood. It is clear, however, that the terminal β1-4 linkage in LNnT prompts this divergent physiological response.

In an effort to determine the contribution of global gene expression to LNT and LNnT metabolism, previously generated RNA-seq data was analyzed in addition to targeting key loci with qRT-PCR. A previous study of *Bifidobacterium breve* UCC2003 concluded that there are overlapping metabolic transcriptional networks with some critical features that are unique between LNT and LNnT metabolism (58). It is noteworthy that *B. breve* evolved the capacity to hydrolyze HMO extracellularly and imports degradation products. There is limited evidence that *B. infantis* is able to do so, which indicates a fundamental physiological difference between the two species. As *B. infantis* captures HMO from its extracellular environment, the complement and expression of transport proteins may catalyze or restrict metabolism of a given HMO species. In this study, the F1SBP Blon_2347 expression differed between the two HMO species, whereas Blon_2177 and Blon_2344 were expressed regardless of the specific HMO. Interestingly, the global transcriptome exhibited a different expression profile for these particular genes. Additional studies are required to resolve the conflict between expression of transporters predicted to be active on type I glycans (i.e., LNT) (16) that were observed to be induced by the type II LNnT. Further characterization of the functional interactions between transport systems and HMO substrates may be essential to address these discrepancies.

The aminosugar residue GlcNAc is a constituent of LNT, LNnT, and all HMOs with a degree of polymerization ≥4. Prior to entering the F6PPK pathway, GlcNAc is processed by two enzymes putatively encoded within the ATCC 15697 genome. This includes GlcNAc-6-P deacetylase (*nagA*; Blon_0882) that deacetylates GlcNAc prior to deamination by glucosamine-6-P isomerase (*nagB*; Blon_0881). *B. infantis* expresses both of these proteins when grown on pooled HMO as reported in a previous study (51). In this study, both LNT and LNnT upregulated these loci supporting the postulate that HMO-bound GlcNAc metabolism contributes to skewing the AA:LA ratio. The elevated AA:LA ratio was observed for pooled HMOs and LNT in a previous study that focused primarily on galactooligosaccharides (47). It is important to note that GlcNAc-bound in HMO may not be fully catabolized through the F6PPK pathway as GlcNAc could serve as a substrate in anabolism including peptidoglycan and other biosynthetic processes (17). Interestingly, ATCC 15697 does not utilize GlcNAc when supplied as sole carbon source in the media in contrast to other *B. infantis* strains. This may be due to a genetic or regulatory variation inherent to the strain. Moreover, hexosaminadase genes were expressed similarly regardless of the particular HMO isomer. These enzymes liberate GlcNAc from galactose through hydrolyzing the β1-3 linkage that is present in both LNT and LNnT.

The terminal galactose, contrastingly, is connected to GlcNAc via a β1-3 linkage in LNT and a β1-4 linkage in LNnT. Thus it was unexpected that a type I glycan-active (e.g. LNT) β-galactosidase (Blon_2016) is downregulated by LNT and not LNnT (*p* < 0.05) (52). This may be due to constitutive expression of other β-galactosidases that cleave the terminal galactose *in vivo*. It is significant that both HMO isomers upregulate two genes that are predicted to feed galactose into the F6PPK pathway. This includes gal-1-P uridylyltransferase (*galT*; Blon_2172) and Uridine 5′-diphospho-glucose-4-epimerase (UDP-glc epimerase, *galE*; Blon_2171) that are localized adjacent to HMO transporters on the ATCC 15697 chromosome. Furthermore, LNnT induces these genes to a greater extent than LNT. This provides mechanistic detail for the physiological differences between LNnT and LNT fermentation.

Interestingly, LNT and LNnT upregulates α-L-fucosidase gene expression despite lacking fucosyl moieties within their respective oligosaccharide structure. Accordingly, LNT strongly upregulates two fucosidases localized to the HMO catabolic cluster. This suggests that there is overlapping regulatory systems or *B. infantis* recognizes LNT and LNnT as signaling molecules to prepare for metabolizing fucosylated HMOs. HMO tetrasaccharides is utilized early in fermenting pooled HMOs prior to fucosylated glycans (19).

Constituent monosaccharides bound in HMO are transformed into substrates catabolized through the F6PPK pathway. Accordingly, LNnT upregulates several genes in this central metabolic pathway likely to satisfy energy demands from a more inefficiently metabolized oligosaccharide. It is interesting that both acetate kinase and lactate dehydrogenase are upregulated by LNnT relative to LNT or lactose. The former is expected given the physiological evidence for increased acetic acid secretion. Lactate dehydrogenase upregulation may be a consequence of fully activating a central metabolic regulon to maintain NAD^+^/NADH homeostasis. There is a significant link between formic acid production and transcriptional processes. LNnT strongly induces formate acetyl transferase (*pfl*; EC 2.3.1.54), which catalyzes formic acid and acetyl-CoA production from pyruvate. In contrast, LNT downregulates this gene as well as pyruvate formate lyase, the latter of which appears to be constitutively expressed during LNnT fermentation. This represents a strong mechanistic association between the LNnT structure and *B. infantis* genomic features to drive the metabolic phenotype.

As bacterial strains of a given taxon may exhibit profoundly dissimilar phenotypes, three additional *B. infantis* strains were examined. *B. infantis* UMA301 exhibits a very similar metabolic response to LNT and LNnT relative to ATCC 15697. Interestingly, these two strains are closely related phylogenetically (30), which suggests that metabolic signature may be a function of phylogenetic divergence for *B. infantis* HMO utilization. Similarly to ATCC15697, LNnT shifts UMA301 metabolism toward formic acid and increases the ratio of acetic acid to lactic acid secretion.

In contrast, UMA300 efficiently utilizes both LNnT and LNT and does not produce formic acid and ethanol on either substrate to the same extent as ATCC 15697 and UMA301. This is likely a function of UMA300 processing LNnT in a efficient manner as LNT, which obviates the need for a metabolic shift. The inefficient HMO-consumer UMA299 exhibits a metabolic response congruent with this unique phenotype among *B. infantis* examined to date (30). The limited capacity to utilize HMO has been attributed to genetic defects within its HMO genomic cluster and provides a control strain linking genotype with metabolic phenotypes. As a result of inefficient growth on LNT and LNnT, UMA299 increases the AA:LA ratio, and formic acid/ethanol production. This is consistent with the hypothesis that diminished capacity for utilizing HMO promotes higher acetic acid concentrations compared to other carbohydrates despite achieving low optical density.

A cell culture approach was used to further develop a model of host-microbial interactions that incorporates inefficient metabolism of specific HMOs. Cell-free supernatants from *B. infantis* HMO fermentations were evaluated for their anti-inflammatory properties on Caco-2 cells. Given the parameters tested, it appears that *B. infantis* reduces inflammation regardless of milk carbohydrate source. Specific anti-inflammatory molecules presented or secreted by *B. infantis* remains hypothetical. Moreover, the extent to which other HMO structures diminish inflammatory outcomes is not understood.

In conclusion, LNT and LNnT utilization increased the AA:LA ratio in all strains. In instances where LNT or LNnT was inefficiently utilized, carbon was shunted toward formic acid and ethanol secretion. A fully integrated mechanistic model underlying this phenotype remains incompletely developed. Thus there is a scientific need to investigate all purified HMO species, additional *B. infantis* strains, as well as other bifidobacterial species to ascertain linkages between HMO structure and physiological responses. This will further refine the metabolic model by which bifidobacteria utilize HMO to colonize the nursing infant colon. In addition to fundamental biological research, there are broad implications to infant nutrition and health. There is accumulating evidence that rationally designing interventions to enhance infant nutrition will require judicious selection of HMOs. This could involve incorporating a single HMO species or a mixture. Clearly a specific HMO tetrasaccharide has different metabolic consequences depending on the *B. infantis* population. There is the potential for strain-level effects to influence the emergent properties of the infant gut microbiome community. Accounting for variation between bifidobacteria, HMO structures, the biology of the infant, and their hosted microbiome communities may enable delivery of precision nutrition and increase impact of the intervention.

## Author contributions

EÖ and DS designed experiments. EÖ performed the experiments and drafted the manuscript through several iterations. DS conceived the study, revised, and approved the manuscript.

### Conflict of interest statement

The authors declare that the research was conducted in the absence of any commercial or financial relationships that could be construed as a potential conflict of interest. The reviewer CS and handling Editor declared their shared affiliation.
