# Supplementary material for: Inefficient Metabolism of the Human Milk Oligosaccharides Lacto-N-tetraose and Lacto-N-neotetraose Shifts Bifidobacterium longum subsp. infantis Physiology
Source: Front Nutr. 2018 May 30;5:46. doi: 10.3389/fnut.2018.00046 (PMC5989456; doi:10.3389/fnut.2018.00046)
Supplement: Supplementary file 1 [file Data_Sheet_1.docx]

Supplementary Material

**Inefficient metabolism of the human milk oligosaccharides**

**lacto-*N*-tetraose and lacto-*N*-neotetraose shifts**

***Bifidobacterium longum* subsp. *infantis* physiology**

Ezgi Özcan^1^ and David A. Sela^1,2,3*^

^1^ Department of Food Science, University of Massachusetts Amherst, MA

^2^ Department of Microbiology University of Massachusetts Amherst, MA

^3^ Department of Microbiology & Physiological Systems and Center for Microbiome Research, University of Massachusetts Medical School, Worcester, MA

***Corresponding author:** David A. Sela: davidsela@umass.edu

# Supplementary Data

**Table S1.** Primers used in this study.

| **Primer** | **Sequence (5’ – 3’)** |
| --- | --- |
| Blon_0393F | CCATGTCCCACCGTTACCT |
| Blon_0393R | CCAGCGACTTCGACATCTTC |
| Blon_0881F | TCGACAACGACATCAACCAG |
| Blon_0881R | AGTAGTCCTTGTGGCGCAGA |
| Blon_0882F | TGGGAGAAGAGCTTCGATGA |
| Blon_0882R | GAACGGGCCTTCCAGATG |
| Blon_0883F | TCCAAGAAGATCGAGGACGA |
| Blon_0883R | CGTAGGGGTTGGTGTACAGG |
| Blon_0884F | GTCGATCCGAACTTCATCCA |
| Blon_0884R | ACCGAGGGCACATAGAGGAT |
| Blon_0885F | GGACTTCTTCGCCTCCATTC |
| Blon_0885R | GGTCATGAGGGTCATGGAGA |
| Blon_2175F | ACCAACACGATGCTCTCGAT |
| Blon_2175R | CCACGAACAGGAACAGGAAG |
| Blon_2176F | CGTCGTGGCATCGATTATCT |
| Blon_2176R | TGTTGTAGCCGGTGAAGGTC |
| Blon_2177F | CGACTACTTCAGCGGACAGG |
| Blon_2177R | ACGGACAGACCGAGGTTCTT |
| Blon_2344F | ACCGGTGAGCACGAATACAC |
| Blon_2344R | TGTAGTCCTTGACGGGATCG |
| Blon_2345F | CTGAACTCGGTGTGGGTGTT |
| Blon_2345R | CCAAGCCAGTGAAGAAGTCG |
| Blon_2346F | GTGGACCTGTCCCTGTACGA |
| Blon_2346R | CCAAGCCAGTGAAGAAGTCG |
| Blon_2347F | GTCCCGAGGCAAGTCCTACT |
| Blon_2347R | AGCTGTCGAGCTTCCTGATG |

**Table S2**. Function of genes described in Figure 5

| **Locus_tag** | **Annotation** | **Function** |
| --- | --- | --- |
| Blon_0248 | alpha-L-fucosidase, GH, family 29 (EC 3.2.1.51) | Fucose metabolism |
| Blon_0268 | GH, family 2 | Enzymatic degradation |
| Blon_0282 | GH, family 13, (EC 3.2.1.1) | Starch and sucrose metabolism |
| Blon_0417 | glucose-6-phosphate isomerase (EC 5.3.1.9) | Sugar metabolism |
| Blon_0426 | alpha-L-fucosidase, GH, family 29 | Fucose metabolism |
| Blon_0459 | beta-N-acetylhexosaminidase, GH, family 20 | Enzymatic degradation |
| Blon_0538 | *galE*, UDP-glucose-4-epimerase (EC 5.1.3.2) | Leloir pathway |
| Blon_0621 | glucan 1,3-beta-glucosidase, GH family 5 | Starch and sucrose metabolism |
| Blon_0625 | beta-glucosidase, GH, family 3 | Starch and sucrose metabolism |
| Blon_0645 | *nanE* N-acylglucosamine-6-phosphate 2-epimerase (EC 5.1.3.9) | Amino sugar metabolism |
| Blon_0732 | beta-N-acetylhexosaminidase, GH, family 20 | Enzymatic degradation |
| Blon_0840 | *ldh*, lactate dehydrogenase, (EC 1.1.1.37) | F6PPK pathway |
| Blon_0881 | *nagB*, glucosamine-6-P isomerase (EC 3.5.99.6) | Amino sugar metabolism |
| Blon_0882 | *nagA*, GlcNAc-6-P deacetylase (EC 3.5.1.25) | Amino sugar metabolism |
| Blon_0883 | extracellular solute-binding protein, family 1 | ABC transporter |
| Blon_0884 | binding-protein-dependent transport systems inner membrane component | ABC transporter |
| Blon_0885 | binding-protein-dependent transport systems inner membrane component | ABC transporter |
| Blon_0900 | type I glyceraldehyde-3-phosphate dehydrogenase (EC 1.2.1.12) | F6PPK pathway |
| Blon_1095 | *tal*, transaldolase (EC 2.2.1.2) | F6PPK pathway |
| Blon_1096 | *tkt*, transketolase (EC 2.2.1.1) | F6PPK pathway |
| Blon_1100 | GH, family 25 | Enzymatic degradation |
| Blon_1368 | ribulose phosphate-3 epimerase (EC 5.1.3.1) | F6PPK pathway |
| Blon_1654 | GH, family 25 | Enzymatic degradation |
| Blon_1714 | *pfl*, pyruvate formate lyase (EC 1.97.1.4) | F6PPK pathway |
| Blon_1715 | *pfl,* formate acetyl transferase (EC 2.3.1.54) | F6PPK pathway |
| Blon_1722 | *xfp*, fructose-6-phosphoketolase (EC 4.1.2.22) | F6PPK pathway |
| Blon_1731 | *ack*, acetate kinase (EC 2.7.2.1) | F6PPK pathway |
| Blon_1740 | GH, family 13 | Starch and sucrose metabolism |
| Blon_1745 | *pyk*, pyruvate kinase (EC 2.7.1.40) | F6PPK pathway |
| Blon_1836 | *eno*, enolase (EC 4.2.1.11) | F6PPK pathway |
| Blon_1843 | beta-N-acetylhexosaminidase, GH, family 3 | Amino sugar metabolism |
| Blon_1905 | beta-glucosidase, GH, family 1 | Starch and sucrose metabolism |
| Blon_2016 | beta-galactosidase, GH, family 42 | Galactose metabolism |
| Blon_2062 | *galK*, galactokinase (EC 2.7.1.6) | Leloir pathway |
| Blon_2063 | *galT*, galactose-1-phosphate uridyltransferase (EC:2.7.7.12) | Leloir pathway |
| Blon_2123 | beta-galactosidase, GH, family 42 | Galactose metabolism |
| Blon_2171 | *galE*, UDP-glucose-4-epimerase (EC 5.1.3.2) | Leloir pathway |
| Blon_2172 | *galT*, galactose-1-phosphate uridyltransferase (EC 2.7.7.10) | Leloir pathway |
| Blon_2173 | *nahK*, N-acetylhexosamine-1-kinase (EC:2.7.1.162) | LNB metabolism |
| Blon_2174 | 1,3-galactosyl N-acetylhexosamine phosphorylase (EC 2.4.1.211) | LNB metabolism |
| Blon_2175 | binding-protein-dependent transport systems inner membrane component | ABC transporter |
| Blon_2176 | binding-protein-dependent transport systems inner membrane component | ABC transporter |
| Blon_2177 | extracellular solute-binding protein, family 1 | ABC transporter |
| Blon_2184 | pgm, phosphoglucomutase, alpha-D-glucose phosphate-specific (EC 5.4.2.2) | Amino sugar metabolism Galactose metabolism |
| Blon_2191 | *rpi*, ribose-5-phosphate isomerase EC 5.3.1.6) | F6PPK pathway |
| Blon_2334 | *lacZ,* beta-galactosidase, GH, family 2 | Galactose metabolism |
| Blon_2335 | alpha-L-fucosidase 2, GH, family 65 | Other glycan degradation |
| Blon_2336 | alpha-L-fucosidase, GH, family 29 | Other glycan degradation |
| Blon_2342 | binding-protein-dependent transport systems inner membrane component | ABC transporter |
| Blon_2344 | extracellular solute-binding protein, family 1 | ABC transporter |
| Blon_2347 | extracellular solute-binding protein, family 1 | ABC transporter |
| Blon_2348 | exo-alpha-sialidase | Other glycan degradation |
| Blon_2350 | extracellular solute-binding protein, family 1 | ABC transporter |
| Blon_2351 | extracellular solute-binding protein, family 1 | ABC transporter |
| Blon_2352 | extracellular solute-binding protein, family 1 | ABC transporter |
| Blon_2354 | extracellular solute-binding protein, family 1 | ABC transporter |
| Blon_2355 | beta-N-acetylhexosaminidase, GH, family 20 | Enzymatic degradation |
| Blon_2411 | GH, family 43 | Enzymatic degradation |
| Blon_2416 | beta-galactosidase, GH, family 42 | Galactose metabolism |
| Blon_2453 | GH, family 13 | Galactose metabolism |
| Blon_2460 | GH, family 36 | Galactose metabolism |
| Blon_2468 | endo-beta-N-acetylglucosaminidase, GH, family 20 | Enzymatic degradation |

**Table S3**. Growth kinetics of *B. infantis* strains while utilizing milk carbohydrates calculated with Wolfram Mathematica 10.3^a^

| **Strains/**  **Carbohydrate source** | **UMA299** | | **UMA300** | | **UMA301** | |
| --- | --- | --- | --- | --- | --- | --- |
|  | **OD_600nm, asym_** | **k (h^-1^)** | **OD_600nm, asym_** | **k (h^-1^)** | **OD_600nm, asym_** | **k (h^-1^)** |
| **Glucose** | 0.955±0.083^B^ | 0.553±0.005^B^ | 0.949±0.055^A^ | 0.580±0.026^AB^ | 0.779±0.066^C^ | 0.301±0.038^C^ |
| **Galactose** | 1.033±0.021^AB^ | 0.590±0.021^AB^ | 1.184±0.209^A^ | 0.753±0.056^A^ | 1.224±0.045^AB^ | 0.625±0.060^A^ |
| **GlcNAc** | 0.423±0.092^D^ | 0.160±0.007^C^ | 0.293±0.080^B^ | 0.154±0.006^C^ | ND | ND |
| **Lactose** | 1.161±0.024^A^ | 0.513±0.009^B^ | 0.525±0.161^B^ | 0.503±0.110^B^ | 1.285±0.050^A^ | 0.561±0.054^AB^ |
| **LNT** | 0.693±0.091^C^ | 0.567±0.053^B^ | 0.998±0.196^A^ | 0.719±0.083^A^ | 1.006±0.167^BC^ | 0.443±0.057^ABC^ |
| **LNnT** | 0.708±0.060^C^ | 0.680±0.057^A^ | 1.300±0.118^A^ | 0.507±0.049^B^ | 0.861±0.042^C^ | 0.395±0.138^BC^ |

^a^The letters indicate significant differences observed between carbohydrates using one-way ANOVA and Tukey’s test (*p*<0.05). ND - not determined.


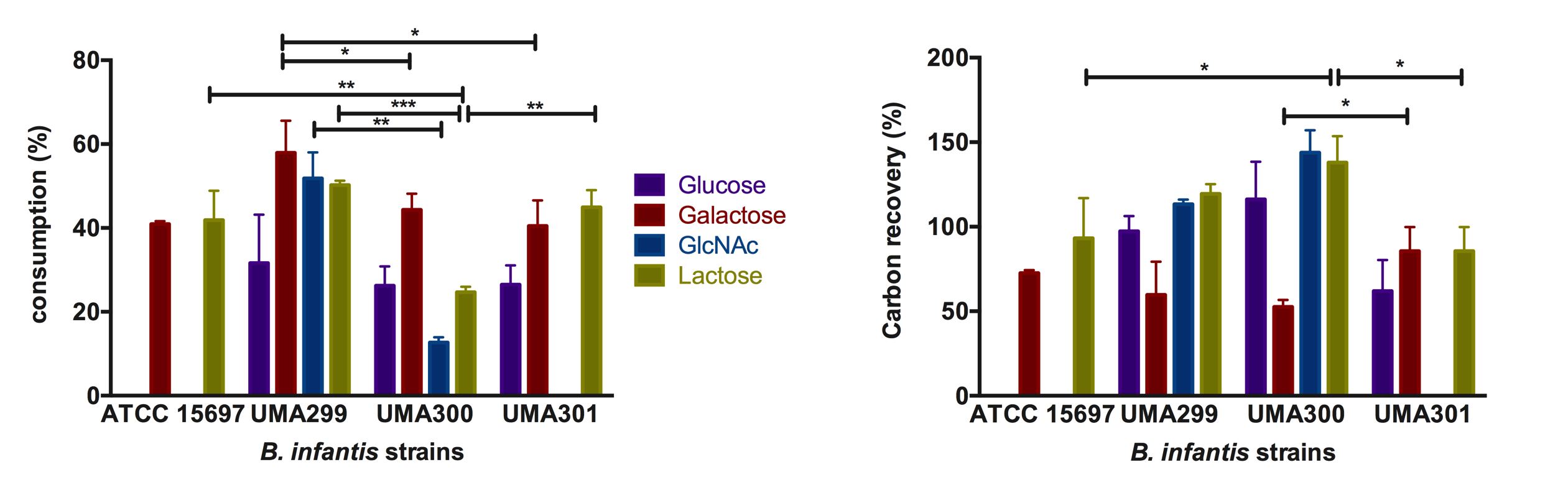


**Figure S1. Consumption of mono- and di-saccharides by *B. longum* subsp. *infantis* strains.** The mono- and di-saccharides consumption in percentages **(A)** and amount of carbon recovered in metabolites in percentages **(B)** of *B. infantis* strains subsisting on mMRS medium containing 2% (wt/v), glucose (purple), galactose (red), *N*-acetylglucosamine (GlcNAc, blue) and lactose (yellow). The data depicts mean ±SD of three independent experiments. The asterisks indicate the significant differences between strains evaluated by one-way ANOVA and Tukey’s multiple comparison. Single asterisk (*) indicates *p*<0.05, double asterisks (**) *p*<0.005, triple asterisks (***) *p*<0.0005. For glucose and GlcNAc, the comparison was made between strains consuming those carbohydrate sources.
